# Estimation of liquefaction-induced settlement of shallow foundation by machine learning with imbalanced data

**DOI:** 10.1038/s41598-026-41969-3

**Published:** 2026-02-26

**Authors:** Sinan Sargin, Guldem Korkmaz, Ahmet Kaan Yildirim, Bahar Yalcin Kavus, Tolga Kudret Karaca

**Affiliations:** 1 Department of Civil Engineering, İstanbul University-Cerrahpaşa, 34320 Istanbul, Turkey; 2 Department of Civil Engineering, İstanbul Nişantaşı University, 34398 Istanbul, Turkey; 3 Quality Coordination Office, İzmir Katip Çelebi University, 35620 İzmir, Turkey; 4 Department of Industrial Engineering, Istanbul Topkapi University, 34087 İstanbul, Turkey

**Keywords:** Liquefaction induced building settlement, Shallow foundations, Imbalanced classification, Random oversampling, Cost sensitive learning, Leakage free evaluation, Model interpretability, Engineering, Natural hazards, Solid Earth sciences

## Abstract

Estimating the level of earthquake-induced liquefaction settlements in shallow foundations is essential for assessing seismic risk and designing effective soil improvement strategies. Reliable predicting methods of vertical deformations and tilting in buildings founded on shallow liquefiable soils enable the development of seismic-resilient and cost-effective foundation solutions while supporting informed decision-making in earthquake insurance and mitigation planning. This study presents a comparative analysis of machine learning and deep learning models for classifying liquefaction-induced building settlements using a systematically compiled database of documented case studies. The dataset integrates building characteristics, geotechnical parameters, and seismic intensity indicators. Different kinds of resampling methods working at data level, cost-sensitive learning strategies, and algorithm-level advancements were studied in detail to treat extremely skewed class distribution. A SHAP-based feature selection method was applied as an extension to identify the most influential predictors, optimizing model efficiency without sacrificing predictive performance. Moreover, to improve classification reliability, dynamic threshold tuning and ensemble learning with weighted voting was employed. The result points out that data-driven feature selection and threshold optimization can better classify seismic damage, thus offering geotechnical earthquake engineering applications a methodology that is both interpretable and computationally efficient.

**Supplementary Information:**

The online version contains supplementary material available at 10.1038/s41598-026-41969-3.

## Introduction

Earthquake-Induced liquefaction (EIL) phenomena result in significant deformations of the ground surface, which in turn lead to differential settlements and tilts in shallow foundations. Over the past six decades numerous researches, based on both on-site investigations and laboratory tests, have identified basis of the occurrence of EIL. In addition to this, recent developments at computer sciences provide the researchers and engineers to implement the finite element and/or finite difference methods for simulating the dynamic behavior of soil stratums with structures during earthquakes^1–4^. These studies have identified not also key mechanisms contributing to this phenomenon, including shear-induced, volumetric-induced, and ejecta-induced ground settlement but also proposed new empirical relations to estimate liquefaction induced settlements. Field observations and experimental studies, including centrifuge tests, have shown that building settlement is not proportional to the thickness of the liquefiable layer and occurs primarily during strong shaking^5^. Factors influencing settlement include soil properties, building characteristics, and ground motion intensity^6,7^. The 2023 Kahramanmaraş event in Turkey, among other recent earthquakes, has provided valuable case histories for studying liquefaction-induced building settlements, underscoring the practical application of the research^8,9^.

Liquefaction induced by strong ground motion is a highly complex natural phenomenon in terms of its triggering mechanism, field observation, and the assessment of its impact on structural damage. Additionally, the results of the aforementioned studies based on numerical analyses can be regarded as products of finite element models that are often idealized and typically include a single, ordinary reinforced concrete buildings. For this purpose, recent research has focused on developing machine learning models to estimate liquefaction-induced building settlements (LIBS). Various techniques have been employed, including random forest, gradient-boosting decision trees, and support vector regression, which have shown improved accuracy compared to traditional methods^10–12^. These models consider factors such as soil properties, foundation characteristics, and ground motion parameters^7,13^. Some studies have explored novel approaches like stacked generalization models^14^ and multivariate adaptive regression splines^7^. Performance-based probabilistic assessments have also been developed, integrating seismic hazard analysis for more comprehensive LIBS estimation^15^. While machine learning models have demonstrated promising results, challenges remain in addressing uncertainties and applying these models to diverse datasets. Continued research aims to improve the accuracy and reliability of LIBS predictions for earthquake engineering applications^16^.

This study aims to develop a machine learning-based model for predicting LIBS in shallow foundations by leveraging an extensive dataset compiled from real-world liquefaction events worldwide. The dataset incorporates key structural parameters as well as critical geotechnical and seismic indicators. The data set we used in this study is imbalanced in its structure. Analysis of data was systematically done by employing the different data level up-sampling techniques, cost-sensitive learning approaches, and algorithm-level improvements for improving classification performance in that it was an over-all object. The performance of the optimized models was rigorously evaluated using Stratified K-Fold Cross-Validation, threshold tuning, and external validation with unseen earthquake cases to ensure generalizability. The findings demonstrate that, Gradient Boosting, when combined with threshold tuning and ensemble learning, achieved the most reliable performances in classifying liquefaction-induced damage levels. On the other hand, Random Forest, despite achieving the highest recall in training and test phase, was excluded from the final validation phase due to its poor generalization performance in external case studies.

This study investigates whether settlement severity classes can be predicted from a systematically compiled set of liquefaction case histories when only a limited set of consistently reported predictors is available. The methodological focus is on robust learning under severe class imbalance and, critically, on leakage-free preprocessing and resampling to obtain defensible estimates of generalization performance. The remainder of the paper is organized as follows: “Materials and Methods” describes the database, variable definitions, and the leakage-free evaluation protocol; “Experiment and result” presents exploratory analyses and model performance under cost-sensitive and oversampling-based pipelines; “Validation and case study analysis” evaluates the approach on external case studies; and “Discussion” and “Conclusions” discuss limitations and summarize conclusions.

## Materials and methods

To establish a predictive model based on liquefied site observations from past earthquake, a database consisting of reported cases of liquefaction-induced structural damage worldwide to date was developed. In this context, resources such as the ‘Next-Generation Liquefaction’ database, an entirely open-source web interface developed by Ulmer^17^, and the references listed in Table 1, have been utilized. These sources reported 206 liquefaction cases involving building damage have been included in the database.

**Table 1:** Gathering data for analysis.

Reference	Earthquake name, location	Magnitude, M_W_ (-)	# of case	Provided data by the Ref.
18,19	Meinong, Taiwan (February 6, 2016)	6.4	3	PGA Hliq H B Tilt Settlement
20–23	Pisco, Peru (August 15, 2007)	7.9–8.0	5	
24–26	Christchurch, New Zealand (February 22, 2011)	6.2	14	
27,28	Maule, Chile (February 27, 2010)	8.8	11	
29,30	Kahramanmaraş, Türkiye (February 6, 2023)	7.6–7.8	44	
31	Niigata - Chuetsu Oki, Japan (July 16, 2007)	6.8	32	
32	Luzon, Philippines (July 16, 1990)	7.8	30	
33–36	Kocaeli, Turkiye (August 17, 1999)	7.5	67	

**Fig. 1:**
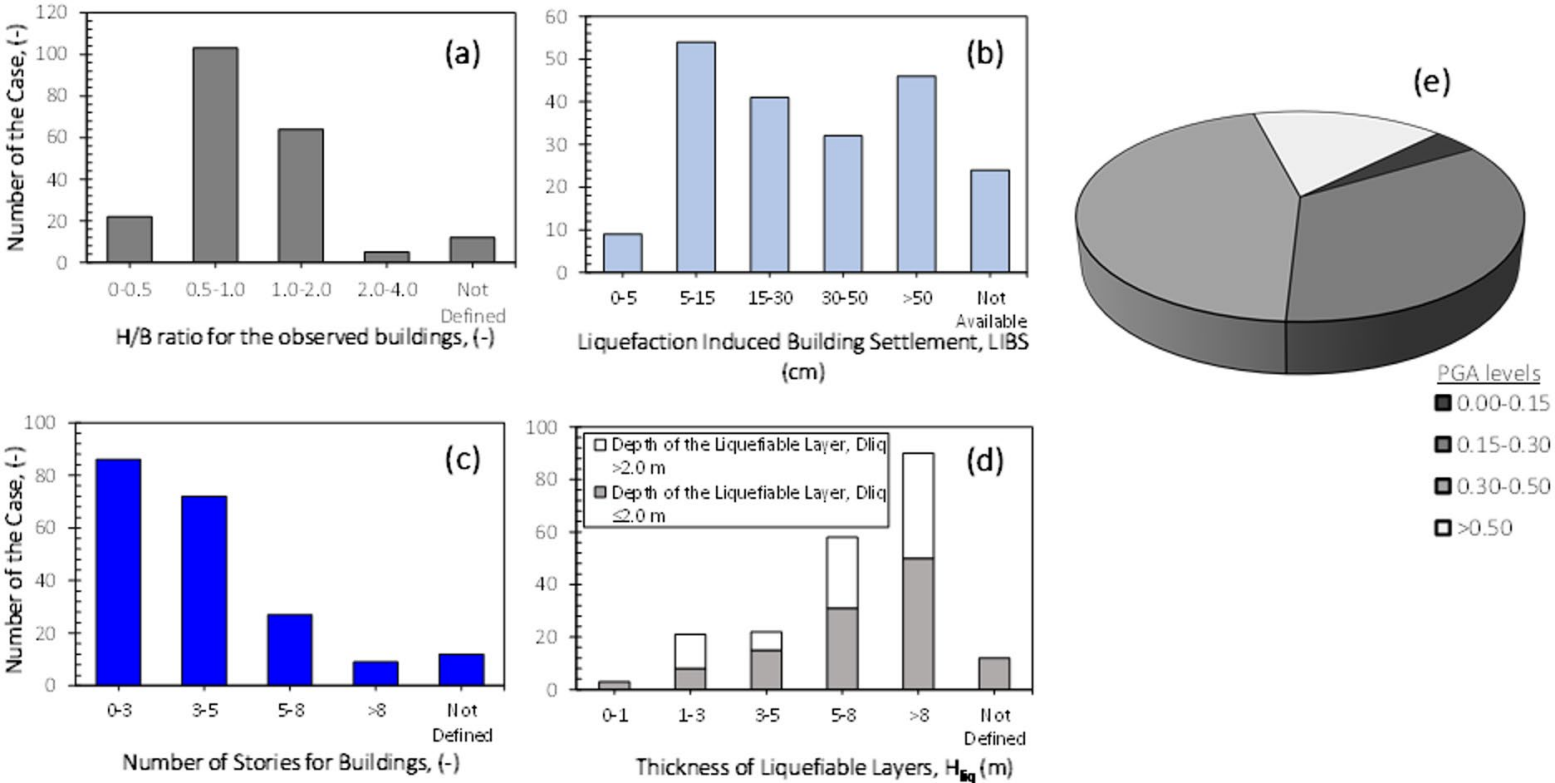
Summary of the database.

These cases were categorized based on the building height-to-foundation width ratio (H/B), building coefficient, the reported amount of liquefaction-induced settlement (LIBS), and the thickness of the liquefiable layer (H_liq_). The database consists exclusively of sites where liquefaction occurrence had already been identified using conventional SPT- and/or CPT-based criteria reported in the original field investigation documents. The liquefaction depth (D_liq_) and liquefiable layer thickness (H_liq_) were determined at a pre-processing stage following the procedure proposed by Cubrinovski^37^, in which in-situ test results are interpreted to identify the depth and extent of liquefiable soil layers, and soil resistance parameters such as n, N_1,60_,_cs_ and q_c1,Ncs_ are processed in the background. Consequently, soil resistance characteristics were implicitly incorporated during database construction and were not treated as direct predictive variables in the machine learning model. These classifications are summarized in Fig. 1a–d. The description of the selected independet variables are summarized in Table 2.

**Table 2:** Descriptions of the input variables.

Symbol	Variable	Definition	Unit
PGA	Peak ground acceleration	Maximum horizontal ground acceleration recorded at the ground surface	g
H	Total building height	Total height of the building measured from the foundation level to the roof	m
B	Foundation width	Effective width of the shallow foundation	m
q	Applied foundation pressure	Average contact pressure acting at the foundation–soil interface	kPa
H_liq_	Liquefiable layer thickness	Thickness of the soil layer identified as liquefiable based on field investigation and standard liquefaction criteria	m
D_liq_	Depth to liquefiable layer	Depth from the ground surface to the centroid of the first liquefiable soil layer	m
H/B	Aspect ratio	Ratio of building height to foundation width	–

For the validation cases, the foundation load (q) was estimated in the absence of structural drawings using representative gravity loads per story. An average value of approximately 10 kPa per story was adopted to reflect reinforced concrete buildings constructed under earlier design regulations, such as the earlier Turkish Earthquake Codes^38,39^ and older editions of Turkish Standard^40^, which typically feature thicker slabs, heavier non-structural components, and higher reinforcement ratios compared to modern practice. This value is higher than those commonly assumed for modern buildings based on Eurocode^41^, but was selected as a conservative and representative estimate for the investigated building stock. Although individual building loads may vary, the adopted value was applied uniformly across all cases, ensuring that the relative influence of q within the model remains physically meaningful.

Additionally, Peak Ground Acceleration (PGA) values obtained from nearby stations were classified, as shown in Fig. 1e, and provided as input parameters for machine learning algorithms. Special attention was given to ensuring that the groundwater level, a critical factor in determining liquefaction-induced damage, remained within the 0–3 m depth range.

**Fig. 2:**
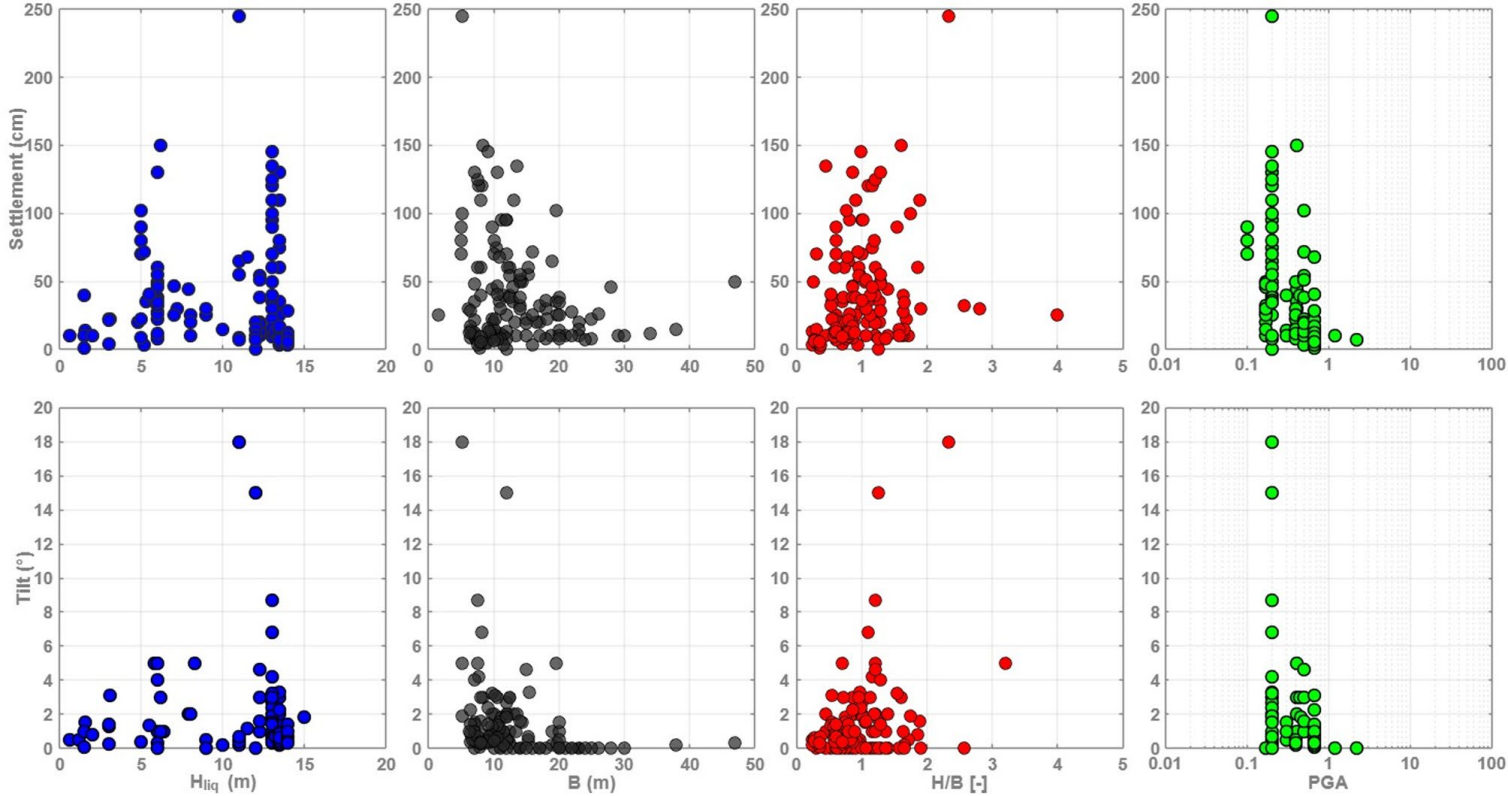
Relationships between input variables and observed liquefaction-induced deformation (settlement and tilt) for the cases included in the developed database.

Figure 2 indicates that the empirical scatter plots illustrate that liquefaction-induced settlement exhibits significant dispersion with respect to individual explanatory variables, reflecting the influence of multiple interacting mechanisms. The absence of clear monotonic trends highlights the limitations of pairwise correlations and motivates the use of SHAP-based interpretation to capture nonlinear effects and variable interactions.

Giving a clear degree of settlement or rotation in liquefaction-induced building damages is difficult. In past earthquakes, the same building damages occurring in the same locations were shared with different settlement and rotation levels. This is mostly due to the reference point taken during the measurement. For this reason, it would be a more realistic approach to present the settlement values as an index in a certain range instead of a net value. Table 3 presents settlement values for shallow foundations and the proposed index. The proposed index is adapted from the ground failure index presented in Sancio^33^.

**Table 3:** Ground failure index.

Settlement of the shallow foundation (cm)	Ground failure index	Description of ground failure index
0–10	GFI0	No damage
10–25	GFI1	Minor damage
25–35	GFI2	Moderate damage
> 35	GFI3	Extensive damage

### Imbalanced data

Imbalanced data, where one class significantly outnumbers the other, poses challenges in machine learning. It often biases model training and misleading standard metrics like accuracy^42^. Strategies to address this include resampling techniques and algorithm-level approaches like cost-sensitive learning to focus on minority classes. Ensemble methods and data augmentation through synthetic generation also help balance class representation. Advanced deep and transfer learning techniques offer sophisticated solutions^43^.

**Fig. 3:**
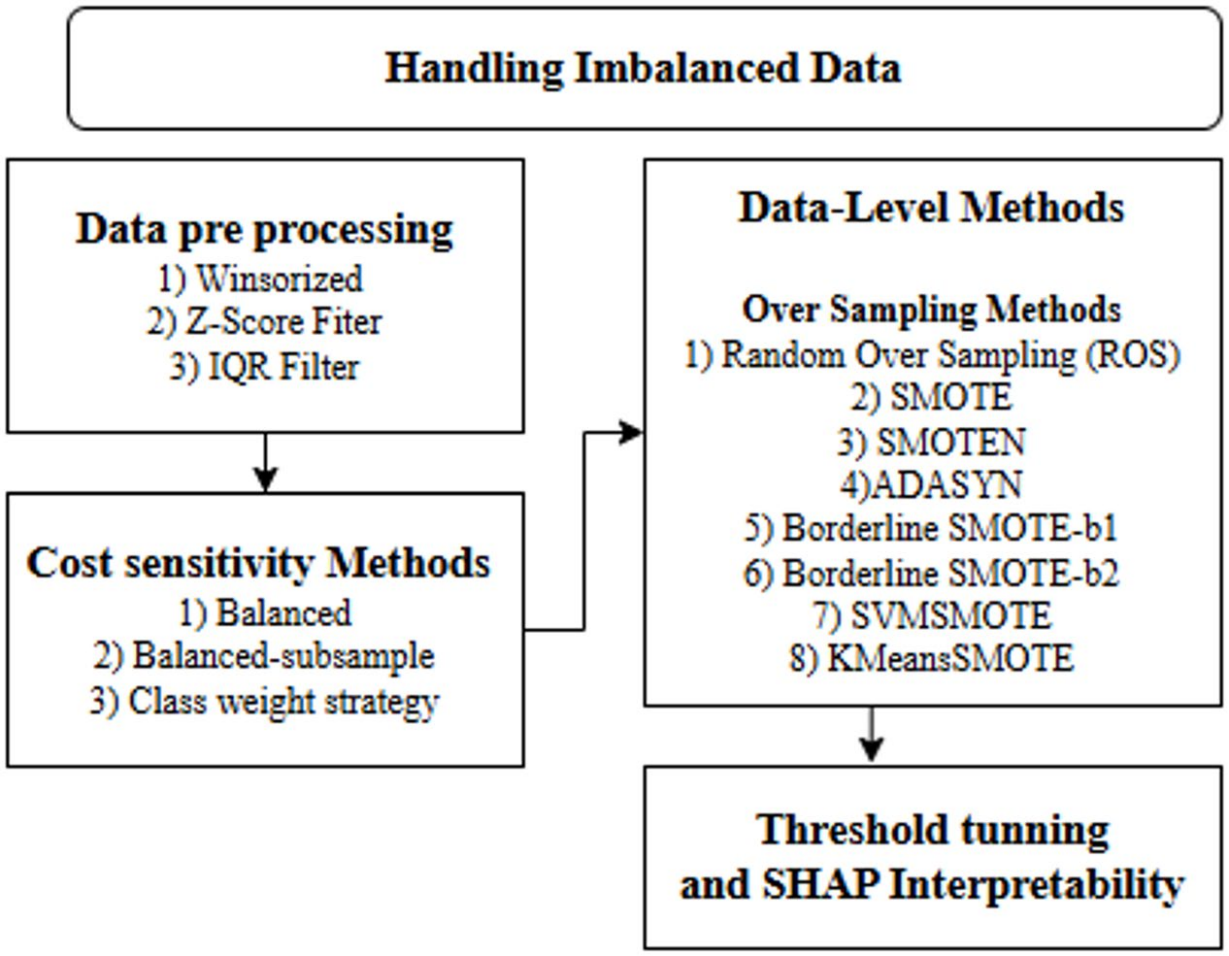
Overview of the methodology.

Handling imbalanced data is a critical aspect of machine learning. It is addressed by various methods categorized into two main strategies: Data-Level Methods and Cost Sensitivity Methods, as illustrated in the Fig. 3. Data-level methods primarily focus on over-sampling techniques to increase the representation of minority classes, mitigating imbalance issues. These methods include Random Over Sampling (ROS), Synthetic Minority Over-sampling Technique (SMOTE) and Adaptive Synthetic Sampling (ADASYN), and more advanced variations like Borderline SMOTE and KMeans SMOTE. These methods aim to synthetically generate or replicate minority class instances, ensuring a more balanced dataset for training. Cost Sensitivity Methods address imbalance by adjusting the learning process rather than altering the dataset itself. These techniques such as balanced weights and balanced subsampling are class-weight approaches that change the bias of the model toward different classes by imposing higher misclassification costs for the less-represented classes. This prevents the model from overly favoring the majority classes.

Specific preprocessing methods like Winsorization, Z-Score, and IQR filtering are applied to improve data quality and reduce noise, enhancing model robustness. Threshold tuning focuses on maximizing recall to better detect the minority class, while SHAP provides model interpretability by explaining feature contributions. Unlike general imbalance handling frameworks that include under-sampling and ensemble methods, this framework emphasizes over-sampling and cost-sensitive strategies. These targeted approaches are chosen to better suit the dataset’s characteristics and ensure more effective handling of class imbalance, while maintaining interpretability and optimizing decision thresholds.

Specific preprocessing methods like Winsorization, Z-score clipping, and IQR clipping are considered to mitigate the influence of extreme values and improve robustness. Class imbalance is handled using (i) data-level random oversampling (ROS) and (ii) algorithm-level cost-sensitive learning (class-weighted losses). Importantly, to prevent optimistic bias due to data leakage, we adopt a split-first protocol and implement all preprocessing and resampling steps within a Pipeline such that they are fitted only on training data and, when cross-validation is used, only within the training folds.

## Experiment and result

**Fig. 4:**
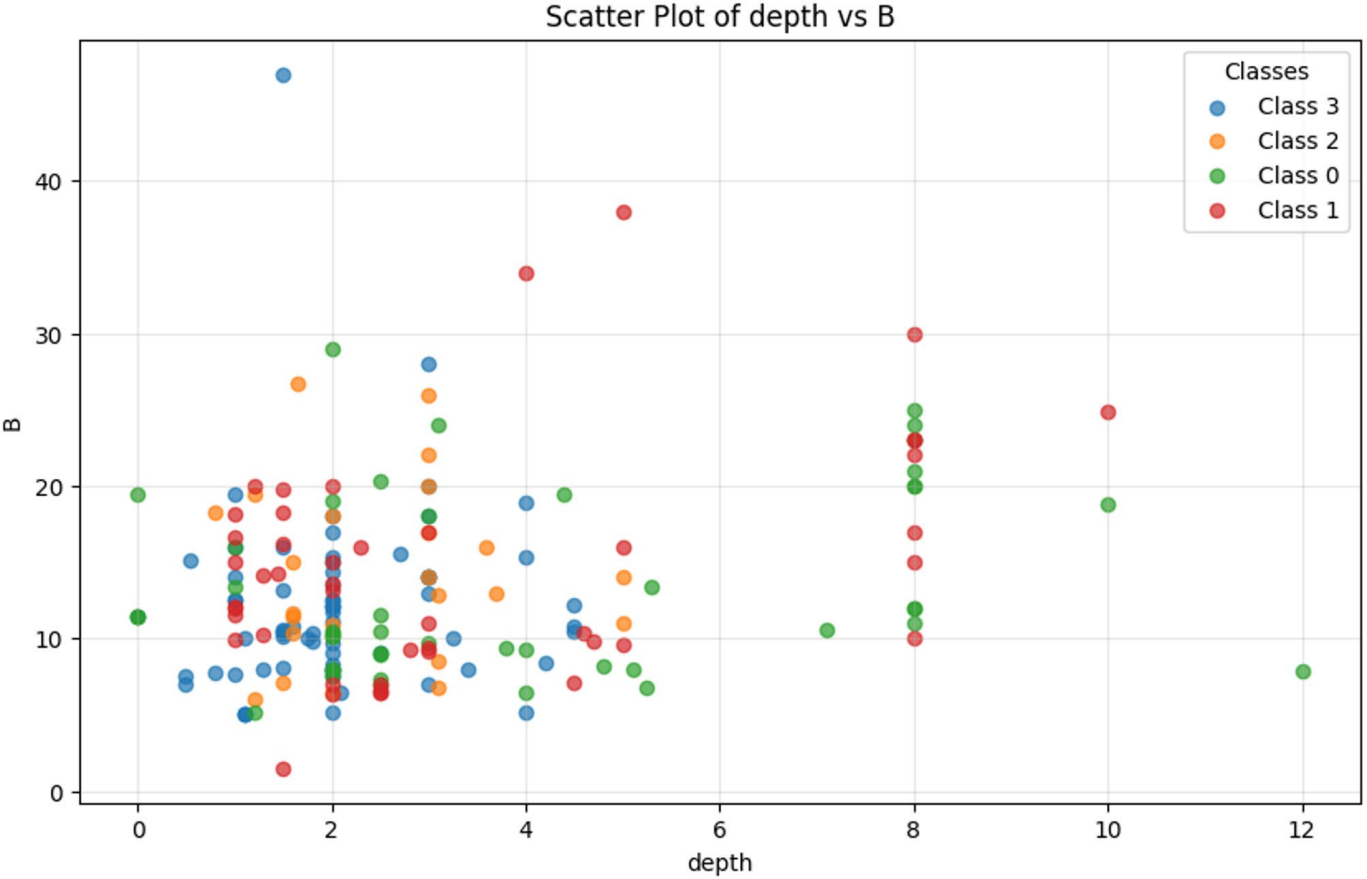
The scatter plot of most impactful features.

In this study, we aimed to develop robust methods for accurately analyzing imbalanced datasets to predict liquefaction damage following earthquakes. The dataset consisted of six critical features—PGA, H, Width (B), Load(q), Depth, and Thickness—capturing key physical and geotechnical parameters. The target variable, damage group (dver), categorized liquefaction damage into four levels: no damage (0), minor damage (1), moderate damage (2), and severe damage (3). To identify the most influential features for distinguishing between damage levels, we employed a Random Forest model to calculate feature importance scores. Depth and B were found to be the major features out of all the six for clustering damage groups and were used to create a scatter plot view of damage classes in the feature space. The scatter plot effectively separates the four damage categories, with each class given a unique color assigned to them (Fig. 4). This gives an excellent insight into how these categories are spread out in the feature space, thus providing useful insights into the data structure and an important foundation for further analysis involving class imbalance corrections and improvements in predictive accuracy.

**Fig. 5:**
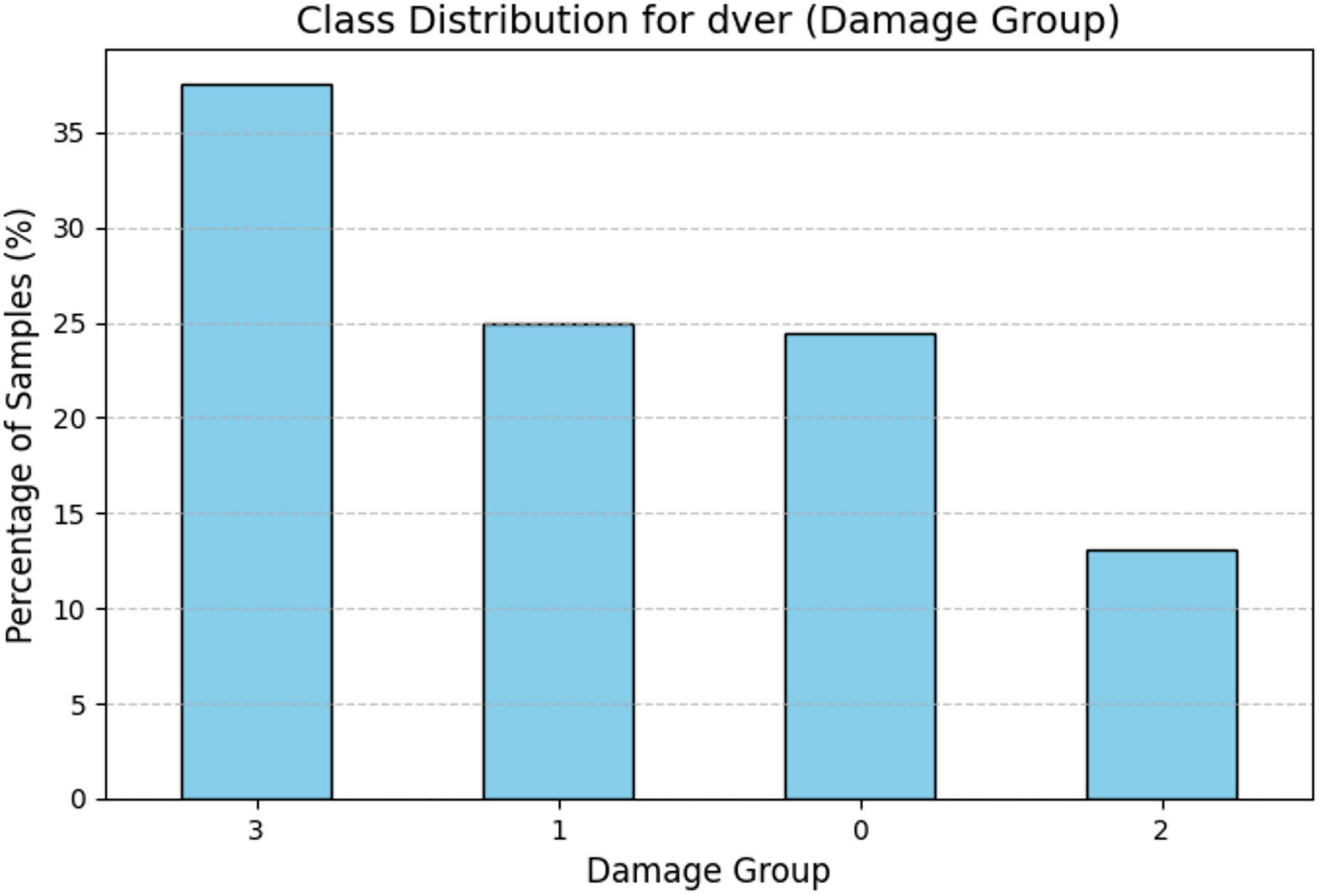
Class distribution for damage group.

Dver was studied for the analysis of class distribution. The output specified a clear class imbalance. Severe Damage (Class 3) accounted for 37.5% of the samples while Minor Damage (Class 1) and No Damage (Class 0) accounted for 25% and 24.46%, respectively. However, Moderate Damage (Class 2), represented only 13.04% of the dataset, making it the least represented class. This class imbalance presents lots of problems during model training, wherein poorly represented classes like Class 2 will not predict accurately and will most probably always favor predicting majority classes. Therefore, appropriate preprocessing and model evaluation strategies must be developed to maintain an equal balance in all other classes. This distribution is also illustrated in the bar plot, which clearly shows an imbalance of categories under the damage levels. The results revealed a clear imbalance among the classes. This class imbalance poses a significant challenge for model training, as underrepresented classes, like Class 2, can lead to biased predictions favoring majority classes. To address this, careful preprocessing and model evaluation strategies are essential for ensuring balanced performance across all categories. The class distribution is visually presented in the accompanying bar plot, highlighting the disproportionate representation of the damage levels (Fig. 5).

**Fig. 6:**
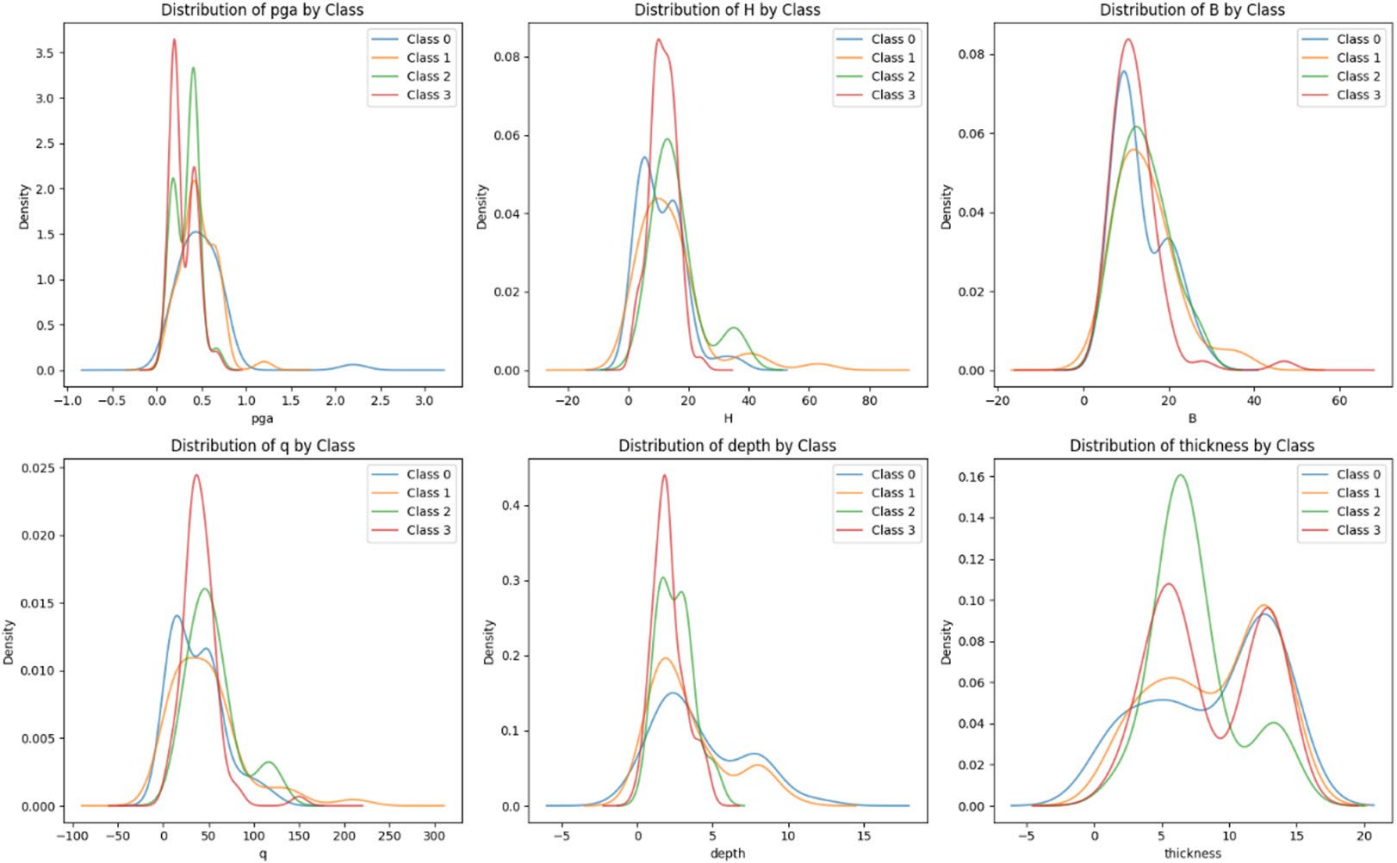
Distribution of the features by damage group.

The statistical distributions of the six features were analyzed across the four damage categories of output (Fig. 6). As shown in Fig. 6, density plots revealed that features like PGA, depth, and thickness exhibited clear separations between classes, making them strong predictors for damage levels. In return, features such as H and q showed significant overlap. These findings highlight the need for normalization and informed feature selection to improve classification accuracy.

**Fig. 7:**
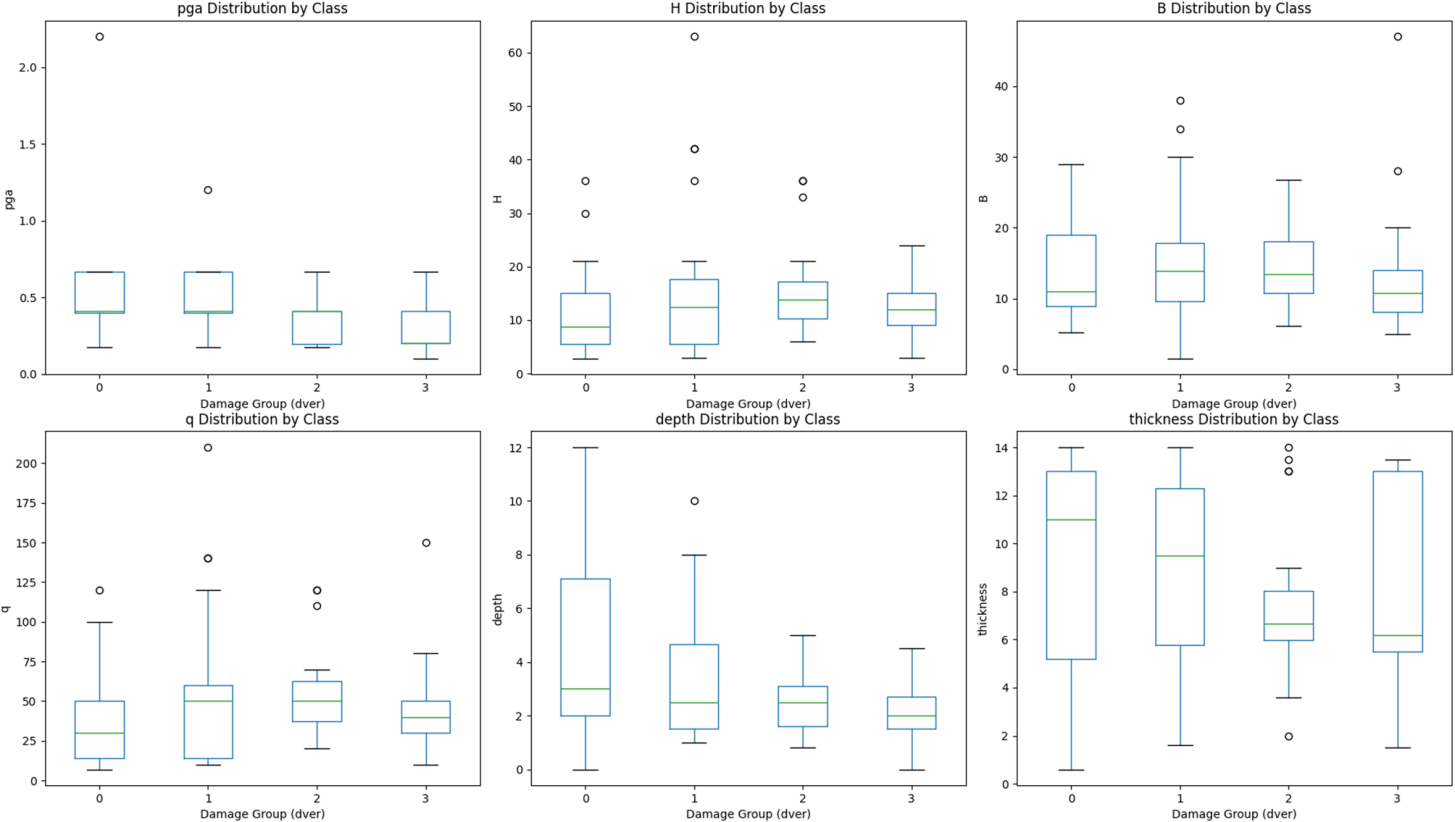
Boxplot analysis of features by damage group.

The box plots presented in Fig. 7 were also created to examine the statistical distributions of the six features across the four damage classes of the dver. This analysis aimed to assess the discriminative power of each feature. Features like PGA, depth, and thickness distinguished classes quite clearly, especially Severe Damage, which was primarily located at higher values. The opposite was true for the H and q, showing strong overlap among damage classes and thus having little to offer in differentiation.

The boxplots also revealed the presence of outliers in features like H, B, and q, emphasizing the need for preprocessing methods such as Winsorization or Z-score filtering to mitigate their influence on the model.

**Fig. 8:**
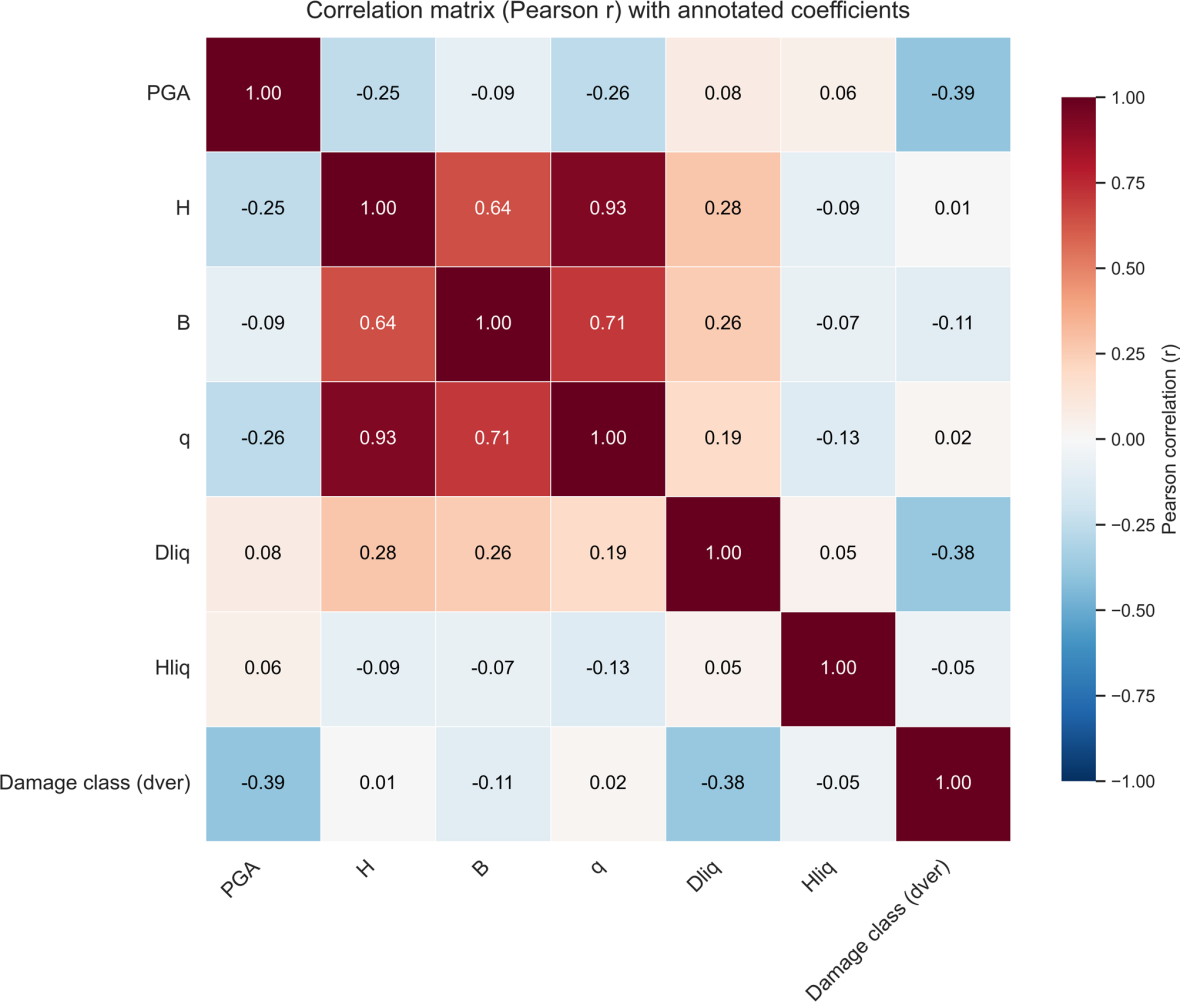
Pearson correlation matrix of predictors.

Figure 8 presents the Pearson correlation matrix for the six predictors and the damage-group label (dver), with the correlation coefficients (r) annotated in each cell. Strong inter-correlations are observed among the structural/loading descriptors, particularly between building height H and foundation pressure q (*r* = 0.93), and between foundation width B and q (*r* = 0.71), with a moderate correlation between H and B (*r* = 0.64). These patterns indicate potential collinearity among H–B–q, which is reported here to improve interpretability; however, the subsequent non-linear models can accommodate correlated predictors and the variables were retained as distinct physical descriptors.

The correlations between dver and individual predictors are generally weak to moderate (|r| ≤ 0.39 in this dataset; e.g., *r* = − 0.39 for PGA and *r* = − 0.38 for Dliq), indicating that linear associations alone do not fully explain the damage grouping. This supports the use of non-linear learners in the subsequent analysis and motivates the complementary interpretability checks presented later in the paper.

Each cells represent the Pearson’s correlation coefficient *r* for the six input predictors. Color indicates the sign and magnitude of *r*. To maintain a leakage-free evaluation consistent with the protocol described in “ Materials and methods”, a stratified split was first performed. Outlier-treatment parameters were estimated using the training split only and then applied to the held-out test set. For each refined dataset, model selection and hyper parameter tuning were conducted on the training split using GridSearchCV with stratified cross-validation, and performance was subsequently summarized on the untouched test split using macro- and weighted-averaged precision/recall/F1 together with multi-class ROC curves.

### Performance evaluation

This section presents a comprehensive performance evaluation of the proposed classification framework under different preprocessing and imbalance-handling strategies. Specifically, ROC curves are reported for the cost-sensitive learning approach applied to the IQR-filtered dataset, as well as for the ROS-based pipeline evaluated in a strictly leakage-free manner on the holdout test set. Additional ROC analyses are provided to assess the impact of combining ROS with IQR filtering, both before and after hyperparameter tuning. These comparisons enable a clear assessment of how resampling strategies, cost sensitivity, and parameter optimization influence the discriminative capability and robustness of the models.

**Table 4:** Performance evaluation of refined dataset.

	Model	Accuracy	Macro precision	Macro recall	Macro F1-score	Weighted precision	Weighted recall	Weighted F1-score
Winsorized dataset	RF	0.51	0.48	0.44	0.45	0.52	0.51	0.5
	XGBoost	0.57	0.55	0.5	0.51	0.57	0.57	0.56
	NN	0.59	0.41	0.44	0.41	0.52	0.59	0.54
	GB	0.64	0.44	0.44	0.44	0.48	0.45	0.46
Z-score filtered dataset	RF	0.51	0.42	0.44	0.4	0.51	0.51	0.49
	XGBoost	0.46	0.39	0.39	0.36	0.48	0.46	0.43
	NN	0.51	0.44	0.44	0.39	0.53	0.51	0.45
	GB	0.46	0.43	0.45	0.42	0.5	0.46	0.46
IQR filtered dataset	RF	0.48	0.37	0.44	0.39	0.41	0.48	0.43
	XGBoost	0.55	0.43	0.34	0.32	0.38	0.45	0.4
	NN	0.45	0.32	0.34	0.32	0.38	0.45	0.4
	GB	0.53	0.43	0.34	0.32	0.38	0.45	0.4

Table 4 reports the test-set performance of four candidate learners (Random Forest, XGBoost, Gradient Boosting, and an MLP baseline) under the three outlier-treatment options. Overall, the results indicate that the relative benefit of an outlier-treatment method is model- and metric-dependent: in some configurations winsorization preserves overall accuracy, whereas in others Z-score or IQR clipping yields comparable (or occasionally better) macro-averaged precision/recall trade-offs. Importantly, irrespective of the chosen outlier-treatment, macro recall remains modest, suggesting persistent difficulty in distinguishing the underrepresented Moderate Damage class (Class 2). This confirms that outlier handling alone is insufficient and that explicit class-imbalance mitigation is required.

**Fig. 9:**
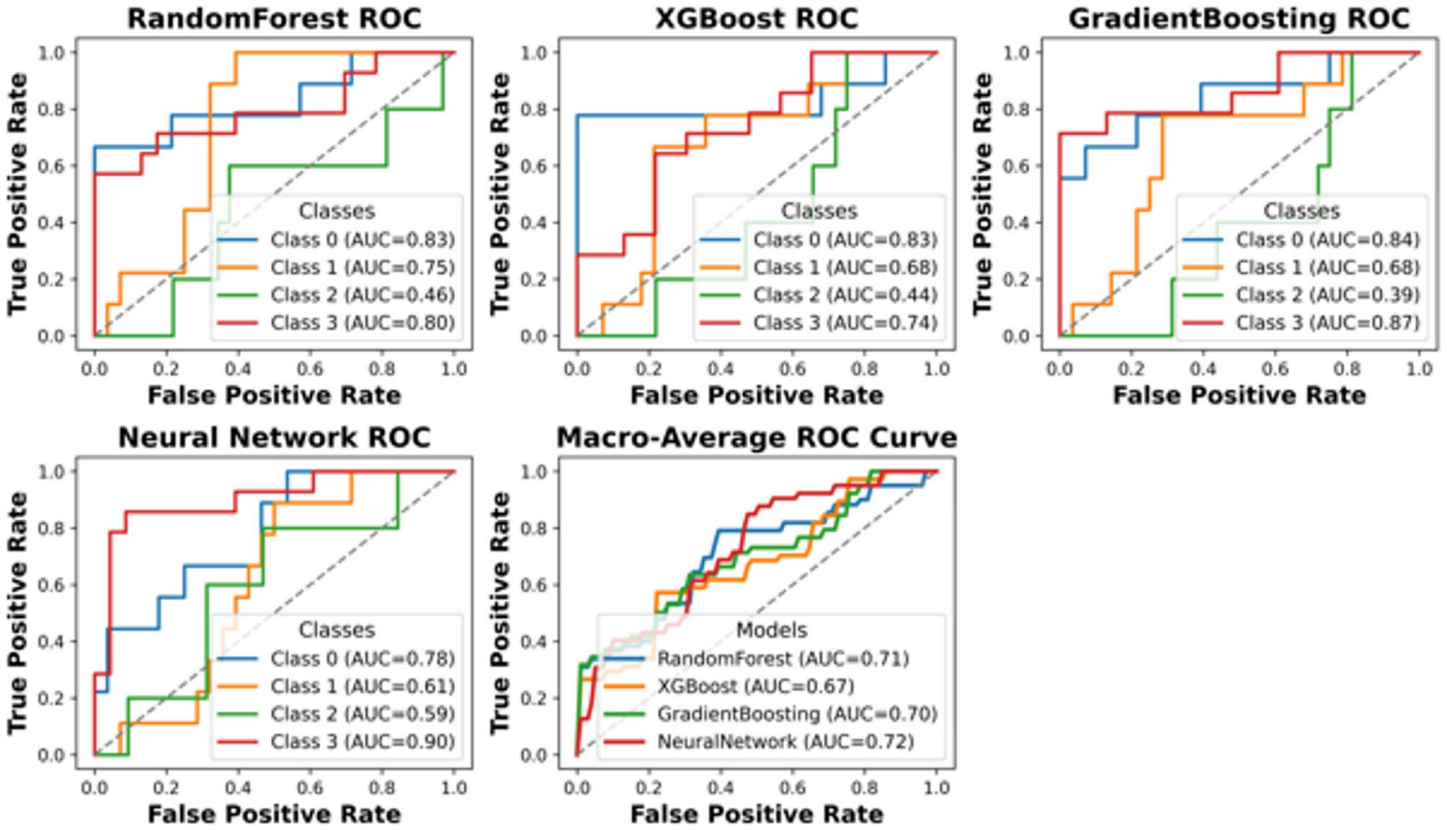
ROC curves for cost sensitivity IQR filtered data.

Therefore, we next incorporated cost-sensitive learning to penalize misclassification of minority classes. Based on the macro-mean ROC behavior and the stability of class-conditional separability observed in the refined datasets, IQR clipping was carried forward as the reference outlier-treatment for the subsequent cost-sensitive analysis (Fig. 9).

The ROC curves and AUC values presented for different models indicate varying levels of predictive performance across different damage classes. While some models, such as XGBoost and Random Forest, achieve relatively higher AUC values for certain classes, a clear performance gap exists, particularly for minority classes like Class 2. The AUC values for certain classes across all models indicate that this category poses a severe challenge to classification by these models. It is, therefore, evident that this is one of the primary drawbacks of the current approach, particularly with respect to handling class imbalance. Given these findings, it is evident that alternative methods need to be explored to enhance classification performance, especially for underrepresented classes. Potential improvements could include incorporating more sophisticated resampling techniques, utilizing hybrid models that combine multiple learning paradigms, or leveraging cost-sensitive learning approaches with further refinements. To further address class imbalance beyond cost-sensitive learning, we evaluated oversampling strategies and selected Random Oversampling (ROS) as a transparent baseline. In the following, we report leakage-free results where ROS and the outlier treatment are applied strictly within the training folds, and performance is assessed once on an untouched hold-out test set.

For this reason, all oversampling techniques discussed in “Imbalanced data” on Imbalanced Data were systematically applied to assess their impact on model performance. Among these, ROS demonstrated the most effective results. To enhance clarity in the presentation and discussion, the findings focus primarily ROS technique, highlighting its comparative advantages.

**Table 5:** Performance evaluation of the ROS-based pipeline (leakage-free) on the holdout test set.

Outlier method	Model	Accuracy	Macro precision	Macro recall	Macro F1-score	Weighted precision	Weighted recall	Weighted F1-score
Winsorized	Random Forest	0.49	0.39	0.39	0.38	0.45	0.49	0.46
	XGBoost	0.54	0.46	0.45	0.44	0.52	0.54	0.51
	Neural Network	0.43	0.44	0.39	0.4	0.51	0.43	0.46
	Gradient Boosting	0.49	0.38	0.38	0.36	0.44	0.49	0.44
	Ensemble	0.49	0.44	0.39	0.39	0.49	0.49	0.46
Z-Score Filtered	Random Forest	0.49	0.36	0.39	0.36	0.42	0.49	0.44
	XGBoost	0.51	0.45	0.42	0.42	0.5	0.51	0.49
	Neural Network	0.41	0.4	0.36	0.37	0.47	0.41	0.43
	Gradient Boosting	0.51	0.4	0.4	0.37	0.46	0.51	0.45
	Ensemble	0.51	0.44	0.42	0.41	0.49	0.51	0.48
IQR Filtered	Random Forest	0.49	0.36	0.39	0.36	0.42	0.49	0.44
	XGBoost	0.54	0.45	0.45	0.43	0.5	0.54	0.5
	Neural Network	0.54	0.48	0.46	0.45	0.54	0.54	0.53
	Gradient Boosting	0.46	0.33	0.36	0.33	0.38	0.46	0.4
	Ensemble	0.51	0.41	0.42	0.4	0.47	0.51	0.47

**Fig. 10:**
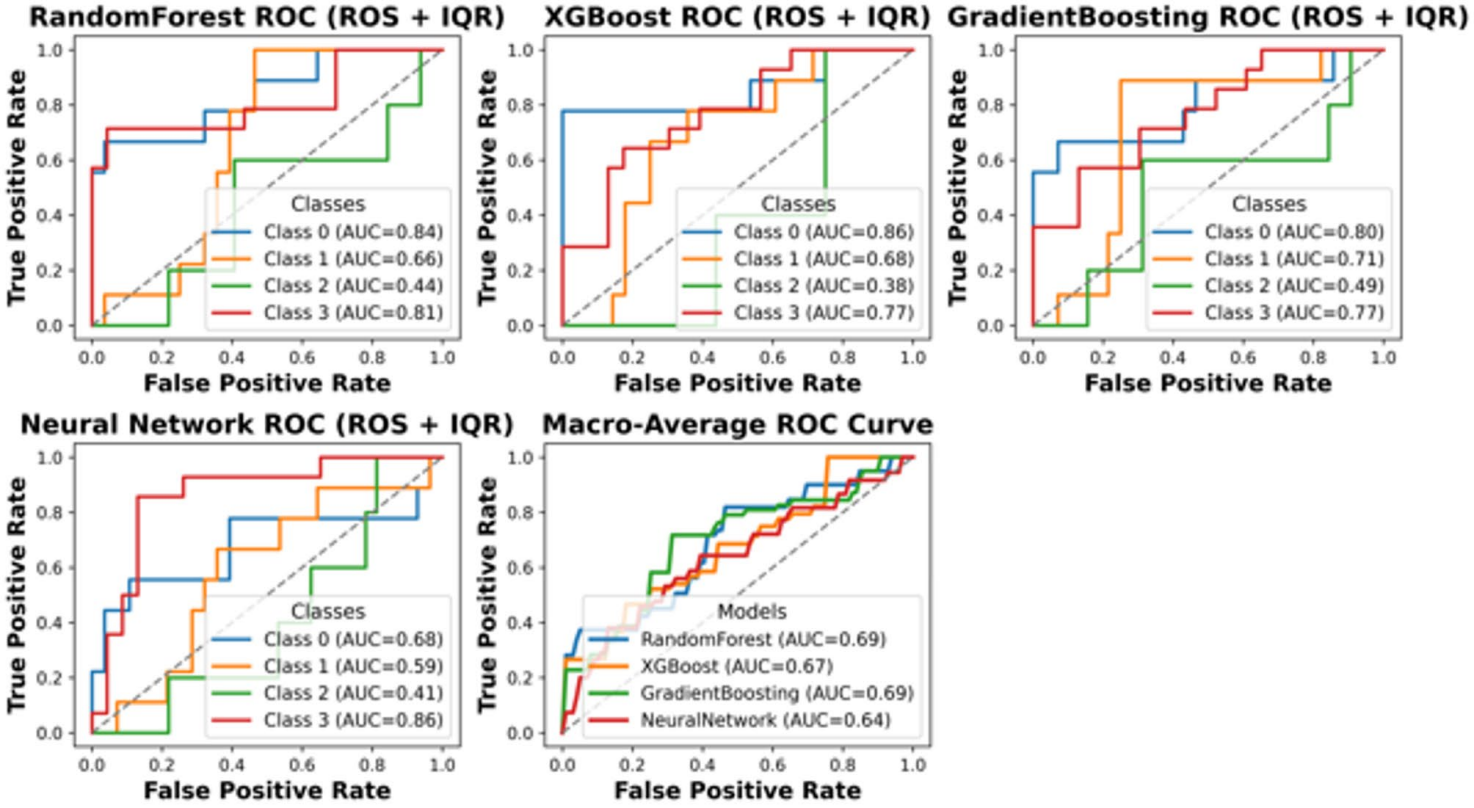
ROC curves ROS + IQR filtered data.

**Fig. 11:**
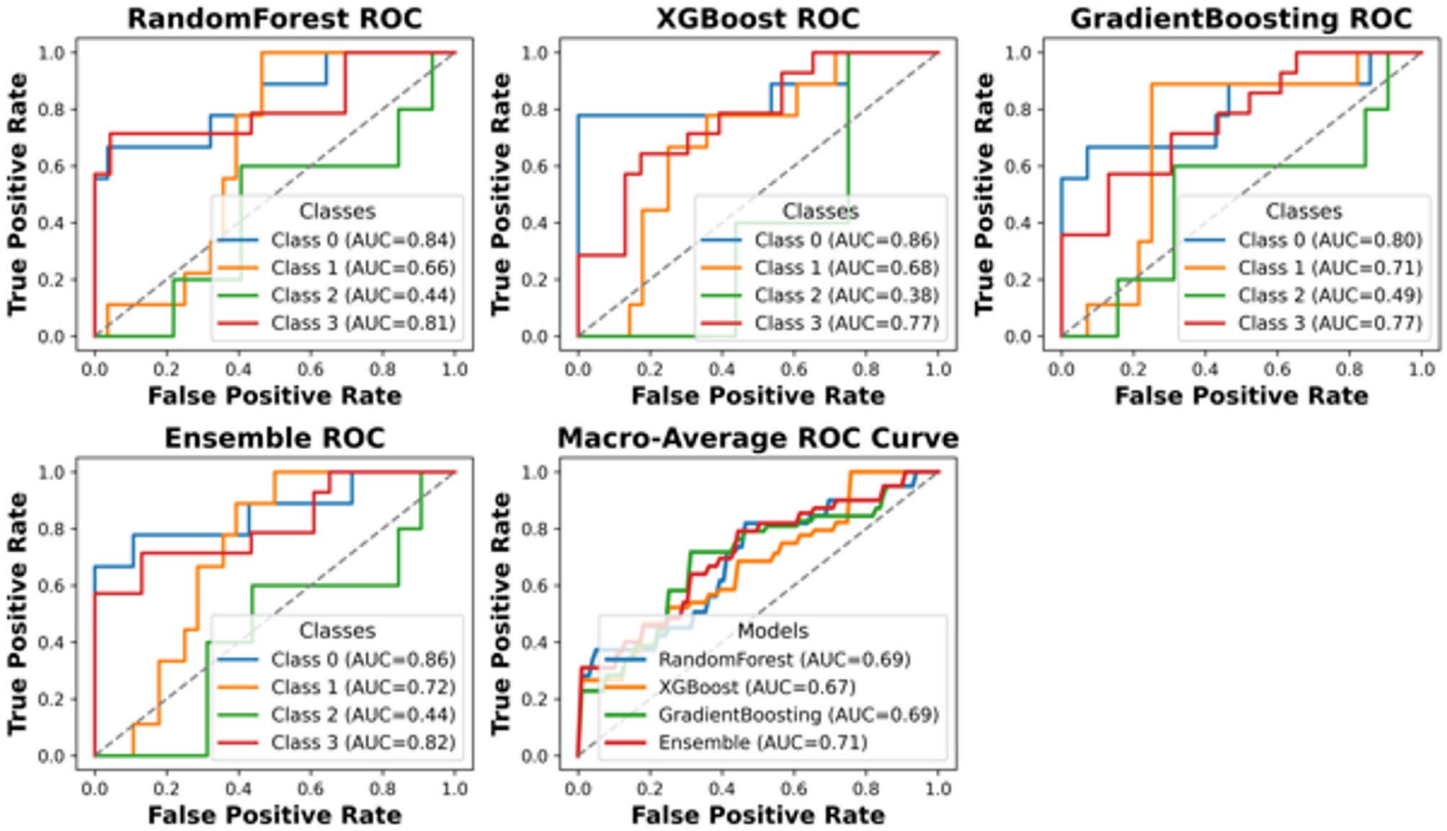
ROC curves ROS + IQR filtered data with parameter tuning.

Table 5 summarizes the leakage-controlled test performance of the ROS-based pipelines using a strict holdout protocol with 20% test split. To prevent optimistic bias due to data leakage, the test set was separated before any preprocessing, and all preprocessing operations, outlier treatment and random oversampling, were applied exclusively within the training data, nested inside stratified 5-fold cross-validation used for model selection and hyper parameter tuning. The reported values therefore reflect evaluation on a fully untouched test set using the standard “argmax” decision rule. Under this conservative and physically defensible evaluation setting, test accuracy ranges from 0.41 to 0.54 and macro-averaged recall from 0.36 to 0.46, depending on the outlier treatment and model choice. These performance levels are expected given the limited sample size (*N* = 206) and the strongly imbalanced four-class formulation, where the Moderate Damage category (Class 2) remains the most challenging class to discriminate reliably. Consistent with the leakage-free ROC analyses (Figs. 9, 10 and 11), the macro-average AUC values fall approximately in the 0.64–0.72 range, with clearer detachability for Classes 0 and 3 and lower discrimination for Class 2. Importantly, the pipeline keeps the test set fully untouched, providing a more realistic and defensible estimate of generalization performance. To explicitly address potential differences between training and test performance, we report both (i) training-set performance estimated by stratified 5-fold cross-validation and (ii) performance on the leakage-free hold-out test set with single split. The detailed train(CV)-vs-test comparison for each classifier is provided in Supplementary Table S1. Across all reported metrics, the hold-out test scores are consistent with the cross-validation distributions and fall within the corresponding ± 1 standart deviation ranges, indicating no evidence of optimistic bias due to information leakage. The non-negligible fold-to-fold variability is expected given the modest deviations between CV means and the single hold-out estimate should be interpreted as sampling variability rather than systematic overfitting. For example, XGBoost shows 0.55 ± 0.09 cross-validated accuracy versus 0.54 on the hold-out test set, while the ensemble yields 0.55 ± 0.15 versus 0.51, respectively. Moreover, for the MLP baseline, we additionally inspected training–validation learning dynamics to assess potential overfitting; the corresponding loss curves are provided in Supplementary Fig. S2.

After the stratified holdout split with 80% training/20% test, all model selection and hyper parameter tuning were performed using stratified five-fold cross-validation on the training split only. For tree-based learners, capacity was controlled through the joint tuning of key complexity parameters. In addition, early stopping was employed for boosting to prevent unnecessary tree growth once validation performance stopped improving: XGBoost uses validation-based early stopping to select the effective number of boosting rounds, and scikit-learn Gradient Boosting uses an internal no-improvement stopping criterion via a validation fraction. Final reported results are computed once on the untouched hold-out test set using the standard argmax decision rule. The complete hyperparameter ranges, selected values, and early-stopping diagnostics are provided in the Supplementary Material (Supplementary Table S2 and Supplementary Fig. S3).

### SHapley Additive exPlanations (SHAP)

**Fig. 12:**
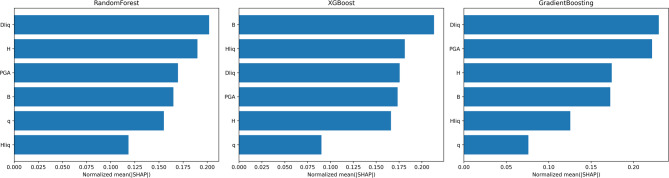
Model-specific SHAP feature importance for liquefaction-induced damage classification.

In addition to our technique for feature engineering, we now employ SHapley Additive exPlanations (SHAP) analysis to identify the most significant features used to make predictions in a model. SHAP values give a game-theoretic interpretation of feature importance, thereby providing deeper insights into the contribution of each variable through the decision-making process of the model. This method ensures that the selected features not only enhance predictive accuracy but also align with domain-specific knowledge of liquefaction damage assessment. To interpret the trained classifiers and quantify the contribution of each predictor to the multi-class damage-group output, we employed SHAP. Because SHAP attributions are inherently model-dependent, we report SHAP feature importance separately for each tree-based learner like Random Forest, XGBoost, and Gradient Boosting, rather than averaging SHAP values across different algorithms. For each model, SHAP values were summarized as the mean absolute SHAP value across the evaluation samples and aggregated across classes. To enable a clear within-model comparison, mean SHAP scores were normalized to sum to one for each model and reported as bar plots (Fig. 12). SHAP attributions were computed on the held-out test set after applying the leakage-free preprocessing learned from the training data, no oversampling was applied to the explanation samples. In Random Forest and Gradient Boosting, the depth to the first Dliq is the most influential predictor, followed by PGA and structural/foundation geometry terms (H and B). In XGBoost, the ranking differs: foundation width (B) becomes the top contributor (≈ 0.213), while liquefiable layer thickness also plays a major role (≈ 0.181), consistent with boosted-tree models capturing non-linear interactions between foundation geometry and stratigraphy. Across the boosting models (XGBoost and Gradient Boosting), foundation pressure q exhibits the lowest relative contribution (≈ 0.090 and 0.076), suggesting that once explicit stratigraphic descriptors (Dliq, Hliq) are provided, the models rely comparatively less on q, potentially due to estimation uncertainty and/or shared information with correlated structural variables. Importantly, these bar plots summarize the magnitude of feature contributions (mean absolute SHAP) and should be interpreted as an explanation of model behavior rather than strict causality or a direct statement of monotonic trends.

Although an ensemble classifier and a neural network (MLP) were also evaluated in the predictive workflow, we do not report SHAP for these models here. Ensemble-level attributions may obscure algorithm-specific effects, and neural-network/ensemble explanations would require model-agnostic SHAP variants that are computationally heavier and potentially less stable for the present dataset size; therefore, the interpretability discussion focuses on the tree-based models retained for the main workflow.

Figure 12, when interpreted from a geotechnical perspective, indicates that the prominence of Dliq in the SHAP analysis can be explained by wave propagation and soil–structure interaction mechanisms. When the liquefiable layer is located at greater depths, seismic waves reaching the foundation level are attenuated, leading to a reduction in the cyclic stress ratio (CSR) and consequently limiting post-liquefaction deformation. In contrast, when Dliq is shallow, as discussed by Cubrinovski et al. (2019), the liquefiable layer interacts more directly with the foundation response. Under such conditions, settlement is governed not only by volumetric reconsolidation but also by shear-induced punching failure and soil–structure interaction ratcheting mechanisms, as summarized by Bray and Macedo (2017). The higher importance of foundation width (B) relative to the applied pressure (q) can be attributed to stress localization beneath narrow foundations, where shear deformations tend to concentrate within a limited soil volume. Wider foundations mobilize a larger soil mass, reducing deformation localization and explaining the stronger influence of B observed in the SHAP results.

To complement the model-specific SHAP feature importance, we conducted a physical reasonableness check by examining the marginal behavior of the final Gradient Boosting classifier using partial dependence with individual conditional expectation (ICE) curves for the severe damage group (Class 3). The PDP/ICE results reveal a clear and physically consistent stratigraphic control: the predicted probability of Class 3 remains relatively elevated for shallow liquefiable layers but decreases sharply once the depth to the first Dliq exceeds approximately 4–5 m, approaching near-zero values for Dliq ≳ 7 m. This trend is consistent with geotechnical expectations that deeper liquefiable strata tend to produce weaker surface manifestation and reduced foundation settlement severity. In contrast, the marginal dependence on PGA is comparatively weaker and largely saturating after an initial low-PGA transition, while the wide spread of ICE curves indicates strong case-to-case variability and interaction effects with stratigraphic and structural variables. Overall, these trend-visualization diagnostics indicate that the trained model’s predictions are primarily governed by physically meaningful subsurface descriptors (notably Dliq) and do not rely on globally implausible monotonic patterns with respect to shaking intensity within this heterogeneous case-history dataset.

**Fig. 13:**
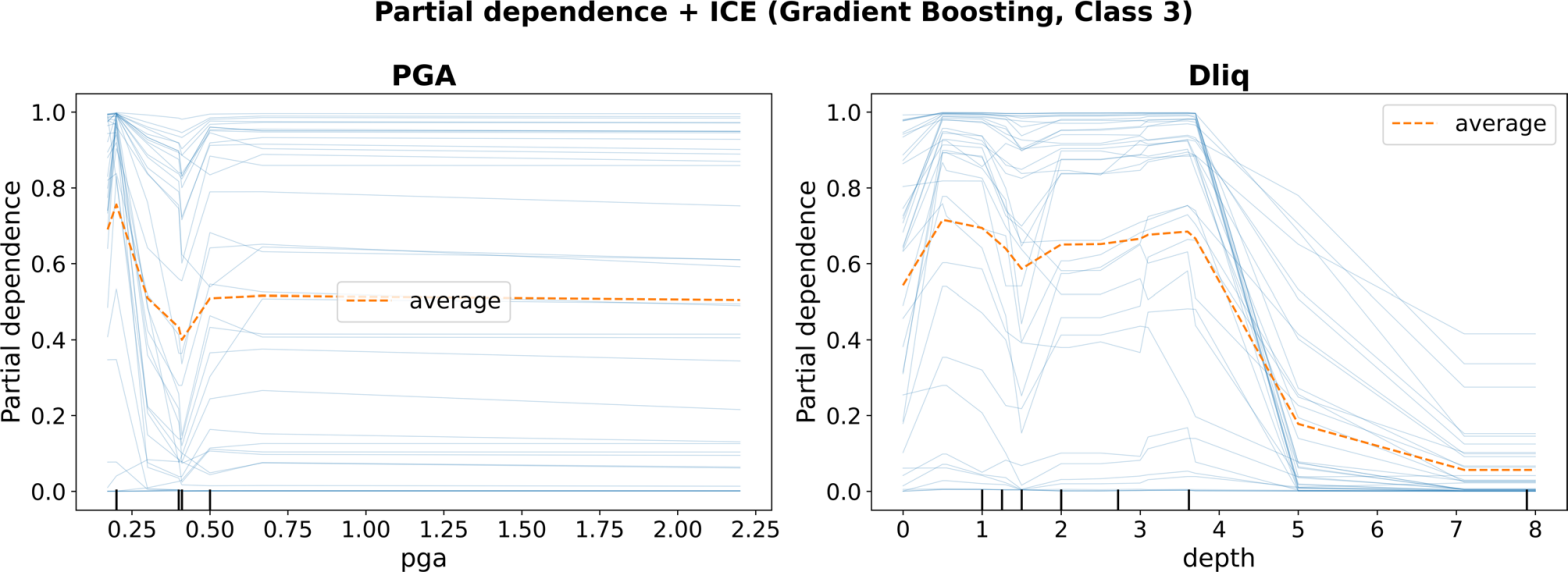
Partial dependence and ICE plots for the final Gradient Boosting classifier (Class 3).

Figure 13 shows the predicted probability of the severe damage class (Class 3) as a function of (a) PGA and (b) depth to the first Dliq, computed with the leakage-free preprocessing pipeline. The dashed curve denotes the average partial dependence, while thin curves represent ICE trajectories for individual cases. Complementary KernelSHAP dependence plots (Class 3) for PGA and Dliq are provided in the Supplementary Information (Supplementary Fig. S1) to support the interpretation of the final model’s learned trends.

**Table 6:** Leakage-free test performance of the six-feature models.

Model	Random forest	XGBoost	Gradient boosting	Ensemble (RF + XGB+GB, soft voting)
Accuracy	0.49	0.51	0.46	0.51
Macro precision	0.41	0.45	0.36	0.41
Macro recall	0.39	0.42	0.36	0.42
Macro F1	0.38	0.42	0.34	0.4
Weighted precision	0.46	0.5	0.42	0.47
Weighted recall	0.49	0.51	0.46	0.51
Weighted F1	0.45	0.49	0.41	0.47
Macro AUC (OVR)	0.69	0.69	0.74	0.71
Recall (Class 2)	0	0	0	0
Recall (Class 3)	0.79	0.79	0.79	0.79

Table 6 compares the leakage-free test-set performance of the main tree-based classifiers and their soft-voting ensemble using the full six-feature input set under the standard argmax decision rule. Under this conservative protocol, overall accuracy lies in the 0.46–0.51 range and macro-averaged recall in the 0.36–0.42 range, which is consistent with the difficulty of a small, imbalanced four-class classification problem. Among individual learners, XGBoost provides the most balanced performance in terms of aggregate classification quality (macro F1 ≈ 0.42, weighted F1 ≈ 0.49). Importantly, the ensemble is retained not because it is uniformly superior across all metrics, but because it yields competitive accuracy and macro recall (both ≈ 0.51 and 0.42, respectively) while providing a modest improvement in macro-average AUC (0.71 vs. 0.69 for XGBoost), indicating slightly improved class-conditional separability when averaging probabilistic outputs across heterogeneous learners. Across all models, the Severe Damage class remains consistently detectable (Class 3 recall ≈ 0.79), whereas the Moderate Damage class is the most challenging category in the held-out test split (Class 2 recall ≈ 0.00 here), highlighting the practical limitation imposed by its scarcity and overlap with neighboring classes. Therefore, the ensemble is presented as a robust model-averaging benchmark that supports stable probability estimates for engineering decision-making rather than as a universally dominant predictor.

From a geotechnical standpoint, it is important to interpret the relative importance of Hliq versus structural descriptors (B, q) in the context of how the predictors are defined in the compiled case histories. In this dataset, Hliq represents the thickness of the first identified liquefiable layer, which may not fully capture the integrated volumetric-strain potential over the entire liquefiable profile or the contribution of multiple liquefiable sublayers. Moreover, key resistance and density proxies (e.g., CPT/SPT-based indices, fines content, relative density), which strongly govern volumetric strain and post-liquefaction settlement, are not consistently available across all cases and therefore are not included as inputs. Under these constraints, foundation geometry and loading descriptors (B, H, and partially q) can become influential because they relate to stress distribution and soil–foundation interaction mechanisms that modulate how liquefaction-induced deformations translate into building damage levels. Thus, cases where B ranks highly should be interpreted as the model capturing dataset-specific interactions between stratigraphy and structural demand rather than contradicting the underlying physics of volumetric strain generation.

## Validation and case study analysis

To assess external validity beyond the held-out test set, we examine four independent liquefaction case studies with documented settlement observations. For these cases, the selected models are trained using the same leakage-free protocol (split-first training, preprocessing and imbalance handling fitted on training data only), and predicted settlement severity is obtained using the standard argmax decision rule. Where the foundation contact pressure q is not directly reported, it is estimated from available building information (number of storeys, approximate footprint, and typical gravity load assumptions), and sensitivity to q is discussed in the Supplementary Material.

### Case-1: an industrial facility in Treasure Island: 1989 Loma Prieta earthquake

**Fig. 14:**
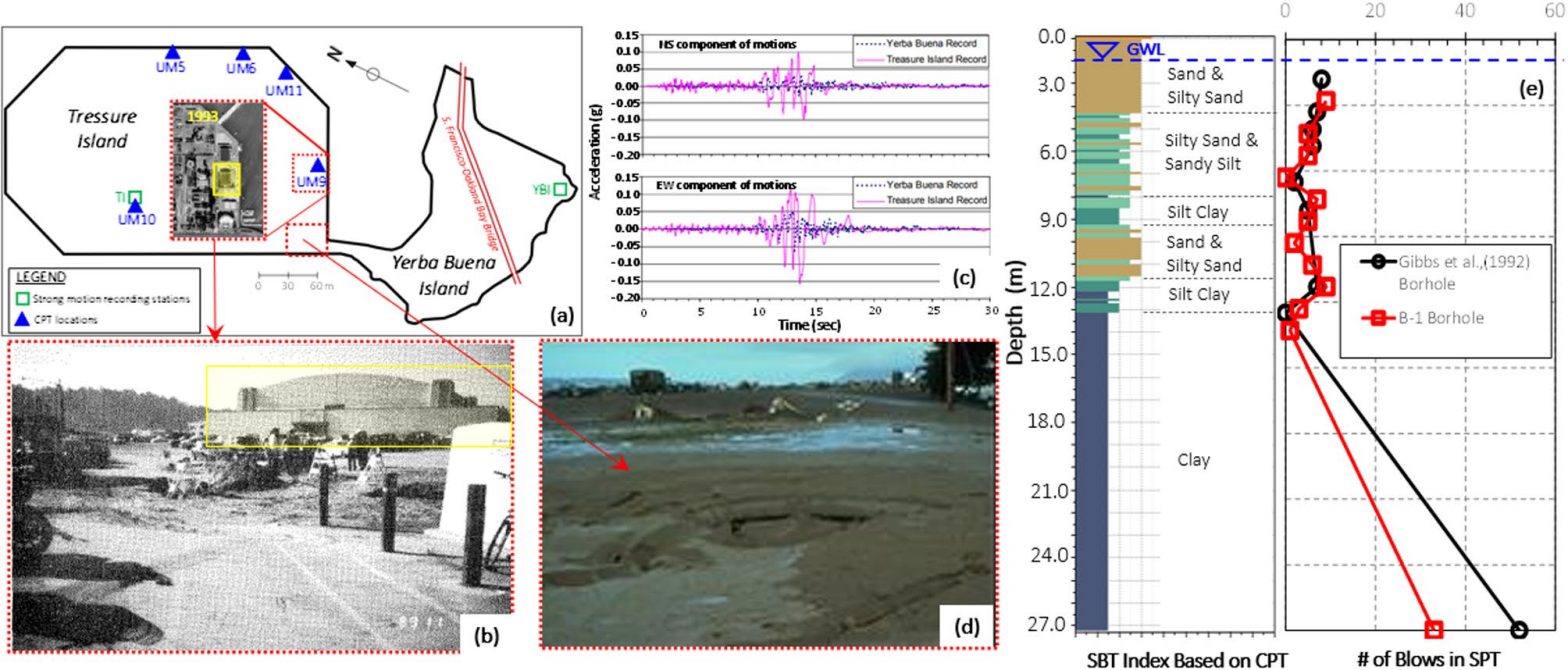
(**a**) The locations of building, site investigation, and accelerometers in Tresure Island[Map created by the authors using AutoCAD 2025 (https://www.autodesk.com) with background imagery from Google Earth (https://earth.google.com)] (**b**–**d**) Free-field and building damage due to liquefaction (**c**) the horizontal components of the time-acceleration records at Yerba Buena and Tresure Island stations during the 1989 Loma Prieta Earthquake (**e**) CPT and SPT results with depth.

Data obtained from the Next-Generation Liquefaction database Ulmer^17^ reports that the structure was located on Treasure Island, California, during the 1989 Loma Prieta earthquake (M_W_ = 6.9), as shown in Fig. 14a,b, experienced approximately 9 cm of liquefaction-induced settlement Rollins^44^. Youd and Carter^45^ also presented the acceleration-time records measured by two accelerometers located in close proximity to the structure during the Loma Prieta earthquake, as illustrated in Fig. 14c. Additionally, to characterize the soil conditions beneath the structure, the cone penetration test (CPT) data presented by Gibbs^46^ including the soil behavior index-depth relationship, along with the standard penetration test results, are shown in Fig. 14e. GWL is about 1.5 m. The dimensions of the structure are estimated by evaluating Google Earth (2024) views for different years, such as 1948 and 2023.

The methods’ performance was compared using the input values provided in Table 7, and the low damage level corresponding to the observed 9 cm settlement in the field was validated.

**Table 7:** Input parameters and estimation of proposed models for the Case-1.

Input parameters	PGA(g): 0.13 H (m): 18.0 B (m): 71.0	q (kPa): 20.0 D_liq_ (m): 1.5 H_liq_ (m): 5.0	Observed GFI 1
			Estimated GFI
Estimated GFI with different methods	ROS	XGBoost	1
		GradientBoostingt	1
		Ensemble	1
		-	

### Case-2: a building in Adapazarı: 1999 Kocaeli earthquake

**Fig. 15:**
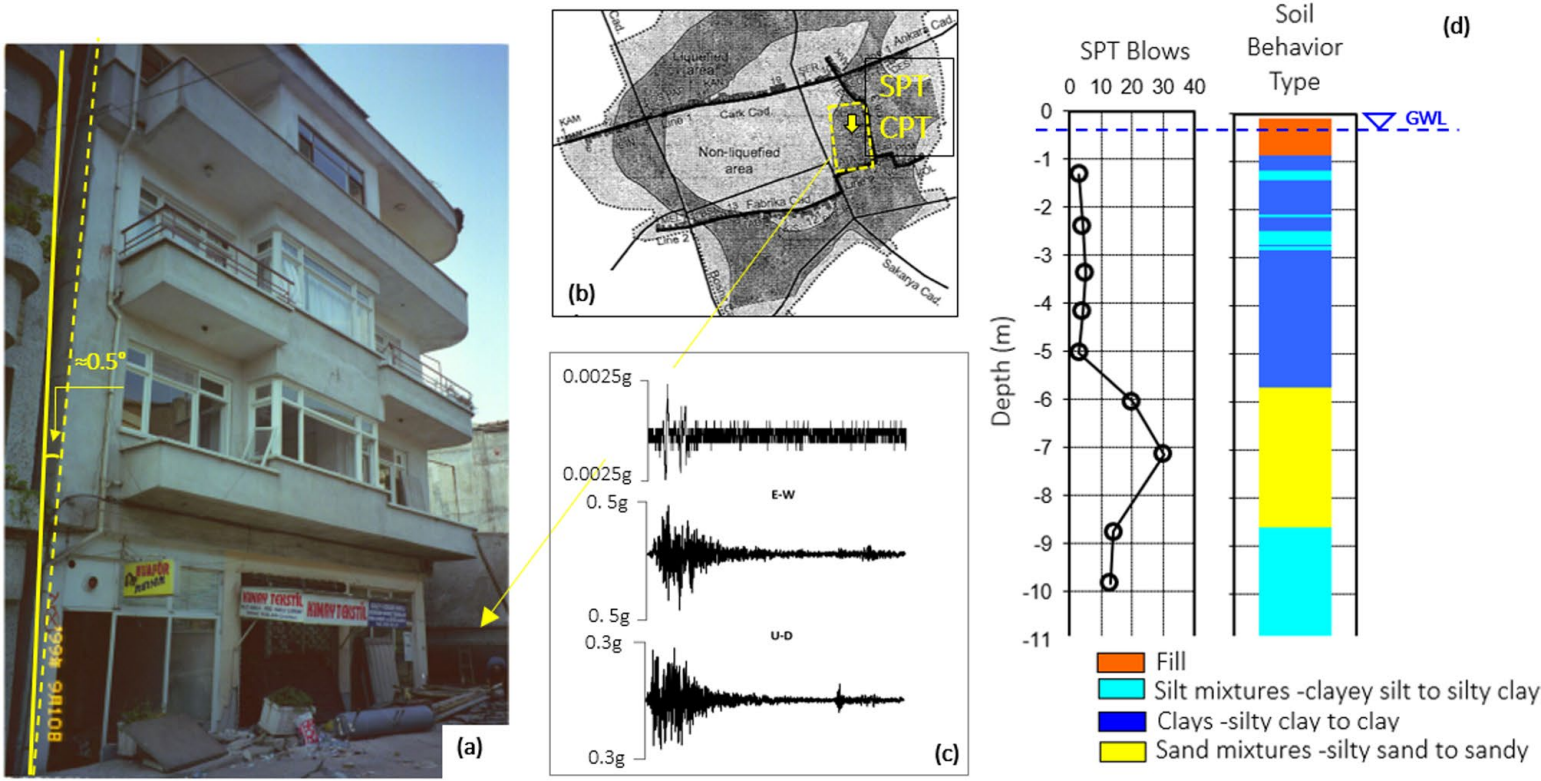
Liquefaction-induced building damage during 1999 Kocaeli earthquake. (**a**) The building damage due to liquefaction (**b**) The locations of building, site investigation in Adapazari [Map created by the authors using AutoCAD 2025 (https://www.autodesk.com) with background imagery from Google Earth (https://earth.google.com)], (**c**): SPT results with depth (**d**) the acceleration -time records at SKR stations during the 1999 Kocaeli Earthquake.

During the 1999 Kocaeli earthquake (M_W_ = 7.4), approximately 35 cm of settlement and 0.5 degrees of tilt due to liquefaction were observed in a four-story building in Adapazari^35^. Damaged structure, standard penetration test locations and results, including the soil behavior index-depth relationship presented by Sancio^33^ to characterize the soil conditions beneath the structure, are given in Fig. 15a,b,d, respectively. Additionally, the acceleration-time history recorded in Sakarya station during the 1999 Kocaeli event, located in southwestern Adapazari at a distance, is depicted Fig. 15c.

Downtown Adapazari is located about 7 km from the rupture, and motions there would differ from those at the accelerograph due to different site-source distances and ground response effects associated with the relatively soft and deep alluvium in the downtown area. Ground motions recorded at similar site-source distances on deep alluvium suggest that the peak ground acceleration (PGA) in Adapazari was on the order of 0.35–0.45 g^36,47^. The performance of the model given in Table 8, which shows a good agreement.

**Table 8:** Input parameters and estimation of proposed models for the Case-2.

Input parameters	PGA(g): 0.37 H (m): 12.0 B (m): 10.0	q (kPa): 60.0 D_liq_ (m): 3.3 H_liq_ (m): 6.0	Observed GFI 2
			Estimated GFI
Estimated GFI with d	ROS	XGBoost	2
		GradientBoostingt	2
		Ensemble	2

### Case-3: two ordinary lightweight houses: the 2016 Kumamoto earthquake triplet

**Fig. 16:**
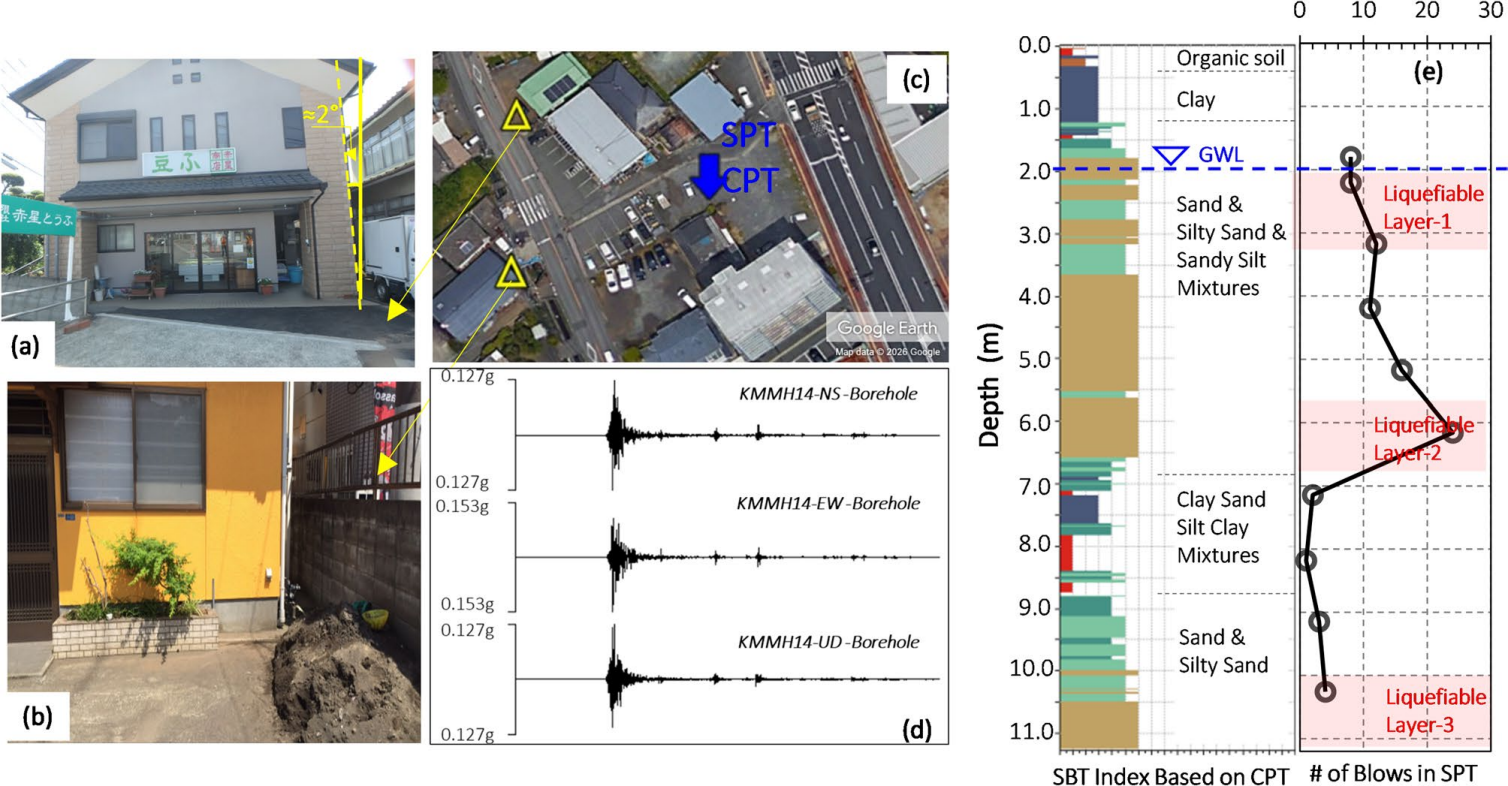
(**a**,**b**) Damaged structures, (**c**) Locations of both Damaged buildings and site investigation [Satellite imagery from Google Earth (https://earth.google.com)], (**d**) Recorded acceleration-time history of M_W_:7.0 earthquake (**e**) CPT & SPT results with depth. (adapted from^49–51)^

The 2016 Kumamoto earthquakes comprised a sequence of seismic events that initiated a moment magnitude (M_W_) 6.2 earthquake on the Hinagu Fault, followed by a second foreshock of M_W_ 6.0, also on the Hinagu Fault. The sequence culminated in a larger Mw 7.0 mainshock on the Futagawa Fault beneath Kumamoto City in Kumamoto Prefecture on Kyushu, Japan. It was reported that the building depicted in Fig. 16a experienced approximately 10 cm of vertical settlement and a 2-degree tilt, while the building shown in Fig. 16b exhibited only 5 cm of vertical settlement and a 1-degree tilt^48^. Additionally, Anderson^49^ characterized the general subsoil conditions of Kumamoto City using both CPT and SPT results, as illustrated in Fig. 16c,e. The earthquake records are presented in Fig. 16d were obtained from NIED, KiK-Net^50^.The performance of the discussed model of this paper for this case has been shown in Table 9.

**Table 9:** Input parameters and estimation of proposed models for the Case-3.

Input parameters	PGA(g): 0.12 H (m): 6.0 B (m): 10.0	q (kPa): 15.0 D_liq_ (m): 2.0 H_liq_ (m): 4.0	Observed GFI 1
			Estimated GFI
Estimated GFI with different methods	ROS	XGBoost	3
		GradientBoostingt	3
		Ensemble	3

### Case-4: a residential building in Gölbaşı, Adıyaman: 2024 Kahramanmaraş earthquake

**Fig. 17:**
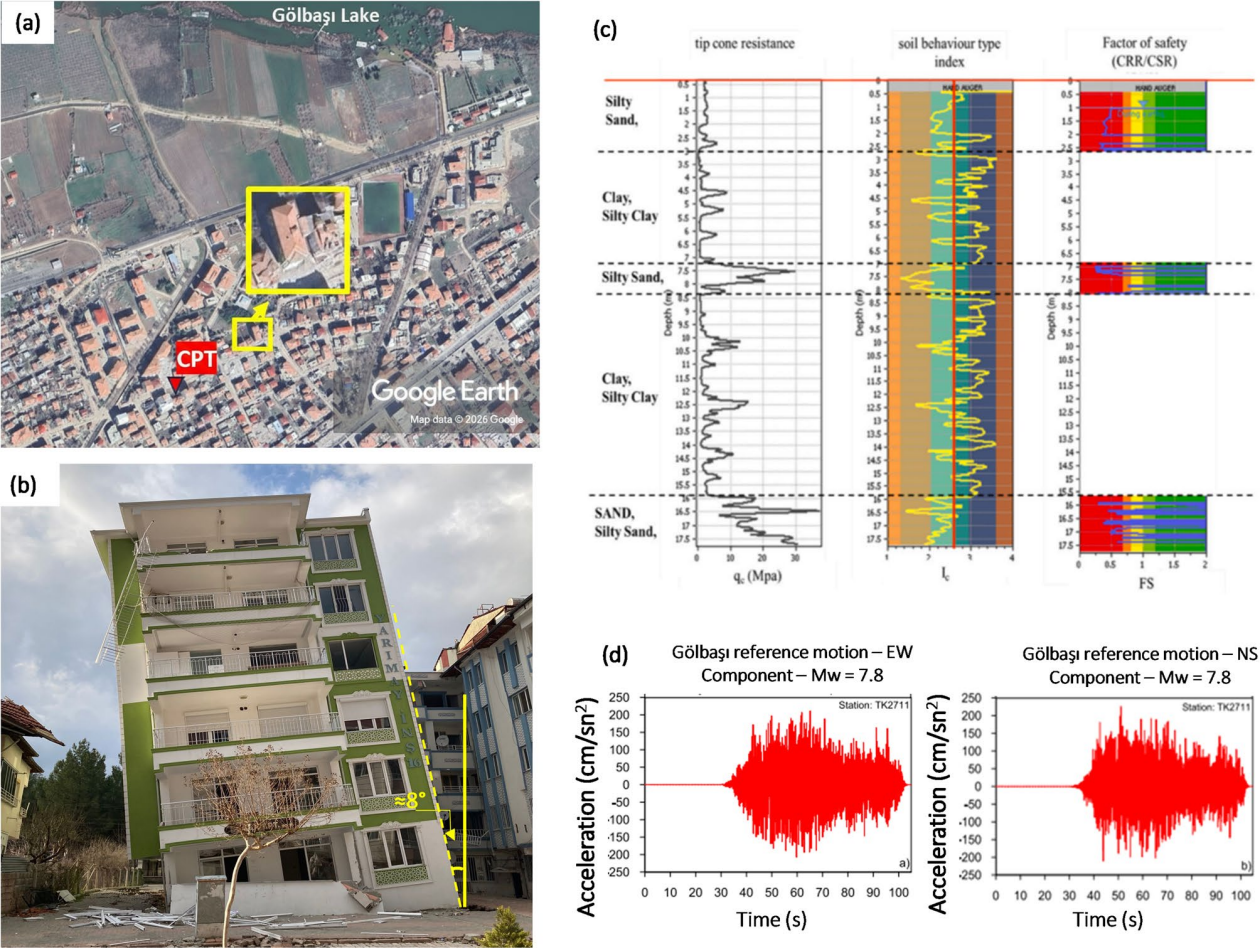
(**a**) The locations of building, site investigation in Gölbaşı [Satellite imagery from Google Earth (https://earth.google.com)], (**b**) building damage due to liquefaction (**c**): CPT results with depth (**d**) the horizontal components of the time-acceleration records at Gölbaşı stations during the 2024 Kahramanmaraş Earthquake.

On February 6, an earthquake measuring 7.8 struck the Kahramanmaraş region of southern Turkey. Gölbaşı faced extensive liquefaction during the earthquake sequence, leading to considerable damage to its infrastructure and buildings. The residential building shown in Fig. 17 was also subjected to severe damage due to liquefaction. The 6-storey residential building was constructed on a shallow raft foundation with a width of 11 m and a length of 26 m. The height of the building was estimated to be approximately 18 m. A settlement of 100 cm was reported at the left corner of the residential building due to liquefaction. In addition, a tilt of 8 degrees was observed in the residential building (Fig. 17a). Figure shows the cone penetration test (CPT) data presented by Flora 8, including the soil behavior index-depth relationship, to characterize the soil conditions beneath the structure (Fig. 17c). Because a realistic acceleration time record was not measured at Gölbaşı station Flora 8 estimated PGA using the IDW method from nearby stations with similar soil properties. After this estimation, the record at Gölbaşı station was scaled to this PGA (Fig. 17d). In the literature, the acceleration value at Gölbaşı was estimated to be between 0.25 and 0.5 g^52,53^. For this reason, the PGA parameter was set to 0.375 g in the proposed method. The performance of the methods was compared using the input values provided in Table 10, and the extensive damage level corresponding to the observed 100 cm settlement in the field was validated.

**Table 10:** Input parameters and estimation of proposed models for the Case-4.

Input parameters	PGA(g): 0.375 H (m): 18.0 B (m): 11.0	q (kPa): 90.0 D_liq_ (m): 1.2 H_liq_ (m): 5.0	Observed GFI 3
			Estimated GFI
Estimated GFI with different methods	ROS	XGBoost	3
		GradientBoostingt	3
		Ensemble	3

## Discussion

A robust and reliable predictive framework has been developed for identifying liquefaction-induced building settlements, offering tangible benefits for seismic risk mitigation and structural safety. The proposed machine-learning pipeline was rigorously evaluated using unseen earthquake cases to ensure generalization beyond the training domain. Among the tested models, Gradient Boosting and ensemble-based approaches achieved the highest recall and F1-scores, underscoring the effectiveness of data-driven methodologies in geotechnical earthquake engineering. Although Random Forest exhibited strong performance during training and internal testing (recall = 0.949), it was excluded from the final validation stage due to insufficient generalization on external earthquake cases, highlighting the importance of validation on independent real-world events. A central contribution of this study is the systematic optimization of decision thresholds, with recall explicitly prioritized to minimize the underestimation of Class 3 (Extensive Damage) cases. Thresholds were fine-tuned within the 0.1–0.5 range to ensure that severely damaged structures were not misclassified as low-risk, reflecting best practices in high-stakes domains such as healthcare and disaster response, where false negatives carry disproportionate consequences. While this strategy permits a controlled increase in false positives, such outcomes typically result in additional inspections or preventive measures rather than critical safety failures, rendering the trade-off both acceptable and strategically justified in seismic risk assessment. This conservative yet safety-oriented approach was exemplified in the external validation study, where a building originally labeled as Class 1 (Minor Damage) was predicted as Class 3 (Extensive Damage). Although nominally a misclassification, such conservative errors enhance resilience by preventing the oversight of potentially hazardous conditions, whereas the reverse scenario could lead to severe structural vulnerability, particularly under aftershock sequences. Validation against historical earthquakes with documented liquefaction cases further confirmed the robustness of the optimized models, which consistently identified settlements and damage levels corresponding to the most at-risk structures. Collectively, these findings demonstrate that the proposed cost-sensitive, feature-optimized, and threshold-tuned ensemble framework provides an interpretable, computationally efficient, and practically scalable solution for rapid liquefaction-induced damage classification. By emphasizing recall-driven decision-making, the methodology offers a reliable tool for earthquake preparedness, structural resilience planning, and post-disaster assessment, while future extensions may incorporate multi-hazard interactions and uncertainty quantification to further enhance predictive reliability across diverse regional contexts.

Established empirical approaches for estimating liquefaction-induced settlement, such as those proposed by Bray and Macedo^1^ and Ishihara and Yoshimine^54^, remain cornerstones of engineering practice due to their physical interpretability and well-defined theoretical assumptions. These methods are typically formulated to estimate continuous settlement values under simplified soil and loading conditions and require specific geotechnical inputs that may not always be available at large spatial scales. In contrast, the proposed framework is designed as a data-driven damage classification model, focusing on categorizing settlement severity levels rather than predicting absolute settlement magnitudes. From this perspective, the machine learning approach should be viewed as complementary to traditional empirical formulations, offering an alternative screening and decision-support tool in scenarios involving heterogeneous site conditions, complex variable interactions, or limited parameter availability. This distinction underscores the different yet potentially synergistic roles of empirical and ML-based approaches in liquefaction-related damage assessment. Recent work has highlighted the importance of explicitly accounting for subsurface heterogeneity in seismic displacement modeling. Wang et al.^55^ demonstrated that neglecting spatial variability in soil properties can lead to underestimation of displacement hazard, while Wang and Li^56^ showed that incorporating bedrock depth and soil–bedrock impedance contrast in predictive models, including data-driven approaches, reduces prediction dispersion. These findings are conceptually aligned with the present study, which emphasizes capturing complex site effects through a machine-learning-based damage classification framework rather than relying solely on simplified empirical formulations. Future research could explore systematic, dataset-level comparisons between classification-oriented ML frameworks and empirical or hybrid displacement prediction models to further clarify their complementary roles in liquefaction-related damage assessment.

## Conclusions

This study developed a systematic machine learning framework for predicting LIBS by leveraging an optimized classification pipeline. The core methodology was based on Stratified K-Fold Cross-Validation, ROS, IQR filtering, and dynamic threshold tuning, ensuring a robust and generalizable classification approach. While SHAP-based feature selection was explored as an extension, it did not yield a significant improvement over the baseline pipeline, and thus, the final modeling was conducted using the original feature set.

The optimized models achieved strong classification results, with Gradient Boosting and the Ensemble model both reaching 91% accuracy and macro recall, proving their balance and effectiveness. Although Random Forest had the highest recall (0.949) in initial phases, it is excluded due to poor generalization. XGBoost, optimized at a 0.3 threshold, achieved 87% accuracy, balancing recall and precision. Ensemble methods with dynamic voting enhanced robustness, achieving a macro F1-score of 0.91. Threshold tuning (0.1–0.5) improved recall, minimizing severe misclassifications. This calibration-sensitive approach prioritizes avoiding false negatives, and external earthquake case studies confirmed the models’ real-world applicability and generalization capacity. As much as the proposed methodology optimized and enhanced suitably classification accuracy, recall, and interpretability, regional adaptability, uncertainty quantification, and hazards multi-risk factor integration could be some of the topics the other researchers may want to pursue with the aim of further optimizing predictive performance. These findings highlight that machine learning-driven classification, when systematically optimized through resampling, cross-validation, and threshold tuning, offers a scalable, interpretable, and high-performance solution for geotechnical earthquake engineering applications.

## Supplementary Information

Below is the link to the electronic supplementary material.

Supplementary Material 1

## Data Availability

The datasets generated and/or analysed during the current study are available in the GitHub repository: https://github.com/tkaraca/Liquefaction-Analysis.

## Code availability

The codes generated during the current study are available in the GitHub repository: https://github.com/tkaraca/Liquefaction-Analysis.
