# Supplementary material for: Estimation of liquefaction-induced settlement of shallow foundation by machine learning with imbalanced data
Source: Sci Rep. 2026 Feb 26;16:11198. doi: 10.1038/s41598-026-41969-3 (PMC13046994; doi:10.1038/s41598-026-41969-3)
Supplement: Supplementary file 1 — Supplementary Material 1 [file 41598_2026_41969_MOESM1_ESM.docx]

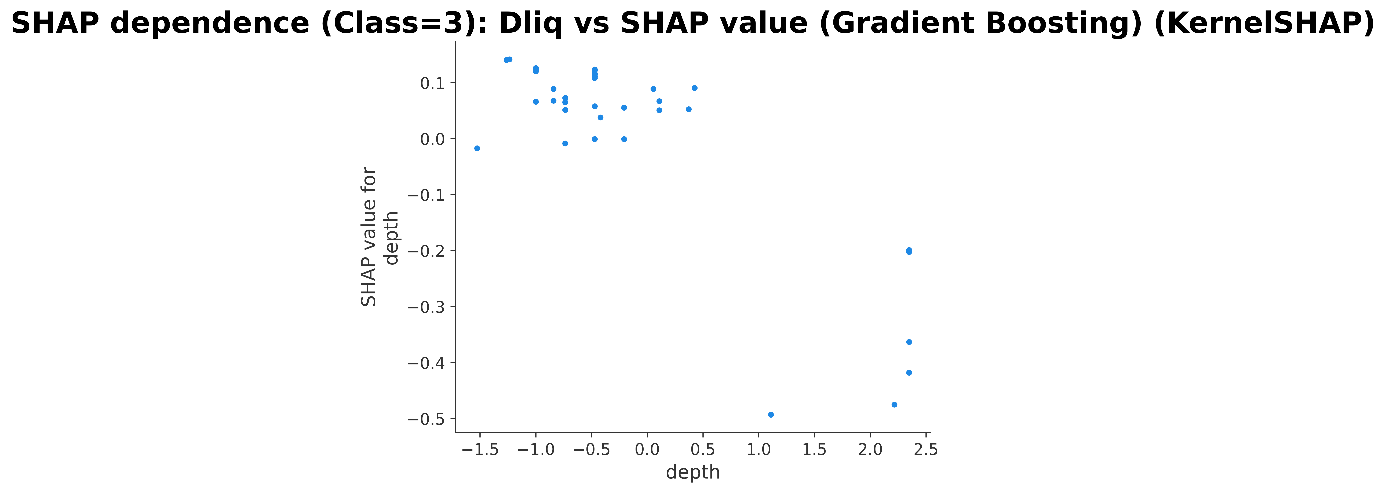


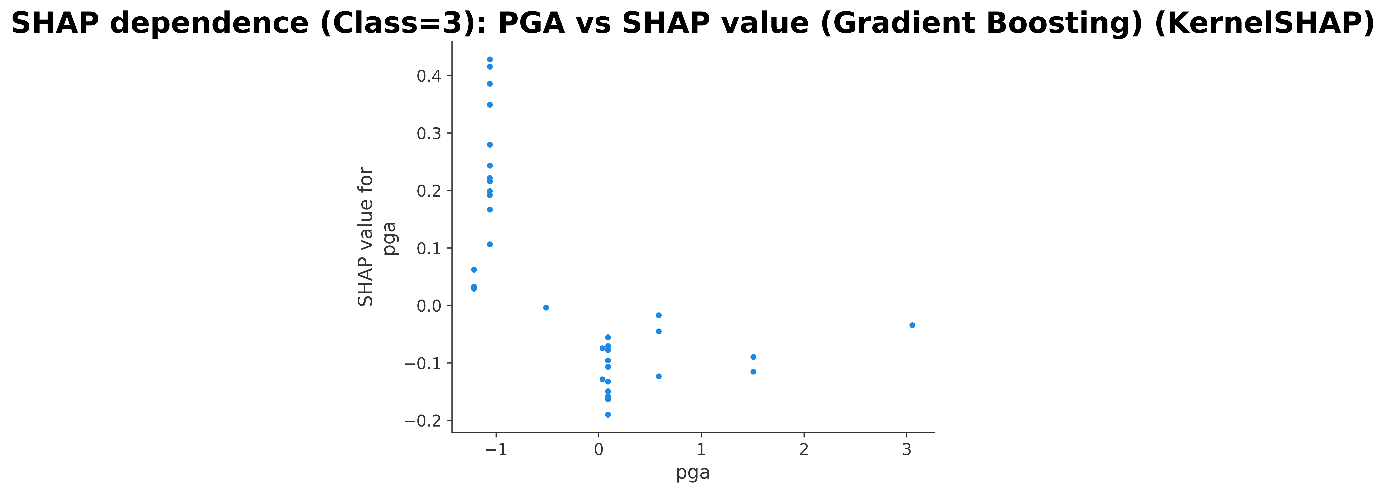


Supplementary Figure S1. KernelSHAP dependence plots for the final Gradient Boosting classifier (Class 3)

Panels show the relationship between (a) PGA and (b) depth to the first liquefiable layer (Dliq) and their KernelSHAP contributions to the predicted probability of the severe damage class (Class 3). Each point represents an evaluation sample; positive SHAP values indicate an increased predicted probability of Class 3 relative to the model baseline. Predictor values on the x-axis are shown in standardized units consistent with the model input preprocessing.

Supplementary Table S1. The detailed train (CV)-vs-test comparison for each classifier

| **model** | **Ensemble**  **(RF+XGB+GB)** | **Gradient**  **Boosting** | **Neural**  **Network** | **Random**  **Forest** | **XGBoost** |
| --- | --- | --- | --- | --- | --- |
| **Train CV (5-fold) Accuracy** | 0.55±0.15 | 0.52±0.17 | 0.40±0.14 | 0.55±0.18 | 0.55±0.09 |
| **Hold-out Test Accuracy** | 0.51 | 0.46 | 0.54 | 0.49 | 0.54 |
| **Train CV (5-fold) Macro Precision** | 0.49±0.21 | 0.44±0.22 | 0.37±0.18 | 0.51±0.21 | 0.54±0.11 |
| **Hold-out Test Macro Precision** | 0.41 | 0.33 | 0.48 | 0.36 | 0.45 |
| **Train CV (5-fold) Macro Recall** | 0.50±0.20 | 0.47±0.21 | 0.38±0.15 | 0.51±0.20 | 0.52±0.12 |
| **Hold-out Test Macro Recall** | 0.42 | 0.36 | 0.46 | 0.39 | 0.45 |
| **Train CV (5-fold) Macro F1** | 0.49±0.20 | 0.45±0.22 | 0.36±0.16 | 0.50±0.20 | 0.51±0.12 |
| **Hold-out Test Macro F1** | 0.4 | 0.33 | 0.45 | 0.36 | 0.43 |
| **Train CV (5-fold) Macro AUC (OVR)** | 0.76±0.13 | 0.72±0.13 | 0.64±0.11 | 0.77±0.12 | 0.75±0.12 |
| **Hold-out Test Macro AUC (OVR)** | 0.71 | 0.69 | 0.64 | 0.69 | 0.67 |
| **Train CV (5-fold) Weighted Precision** | 0.54±0.18 | 0.49±0.19 | 0.43±0.18 | 0.55±0.20 | 0.56±0.11 |
| **Hold-out Test Weighted Precision** | 0.47 | 0.38 | 0.54 | 0.42 | 0.5 |
| **Train CV (5-fold) Weighted Recall** | 0.55±0.15 | 0.52±0.17 | 0.40±0.14 | 0.55±0.18 | 0.55±0.09 |
| **Hold-out Test Weighted Recall** | 0.51 | 0.46 | 0.54 | 0.49 | 0.54 |
| **Train CV (5-fold) Weighted F1** | 0.53±0.17 | 0.50±0.18 | 0.40±0.16 | 0.54±0.19 | 0.54±0.10 |
| **Hold-out Test Weighted F1** | 0.47 | 0.4 | 0.53 | 0.44 | 0.51 |


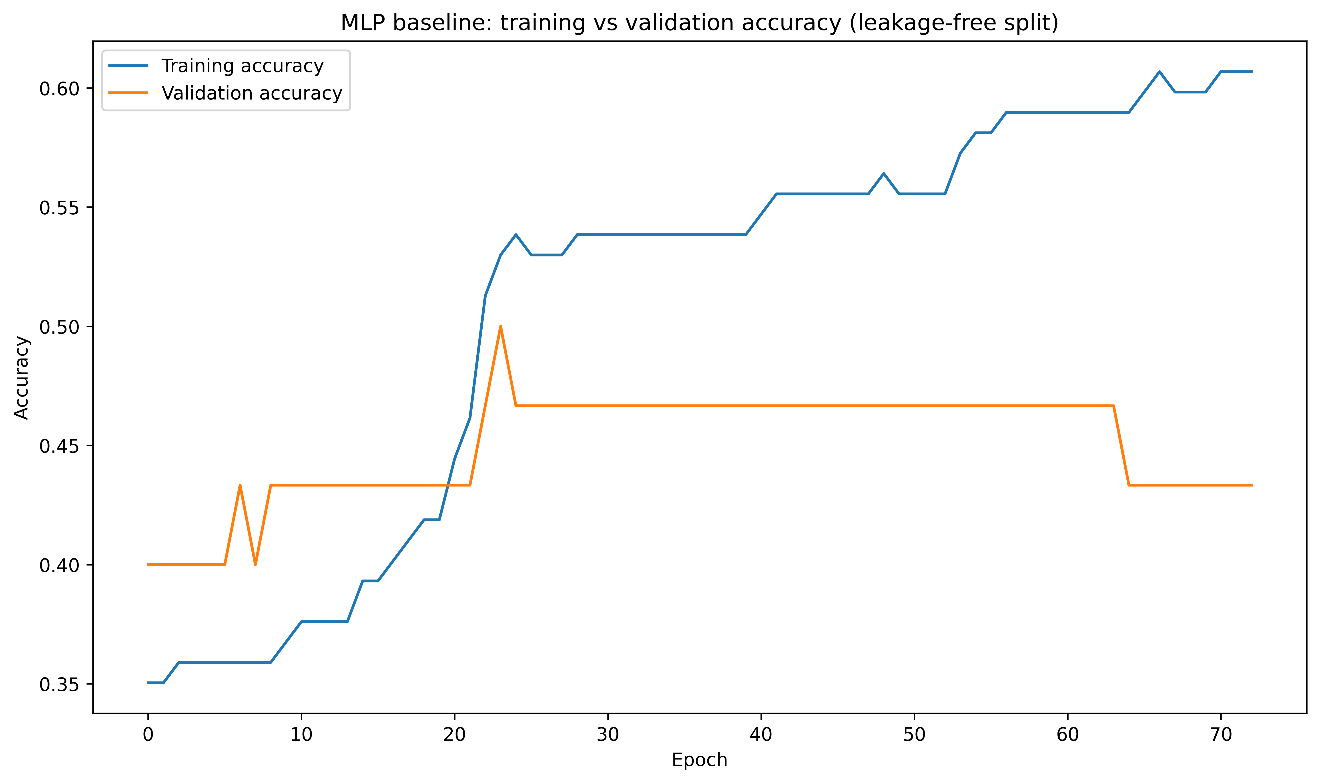


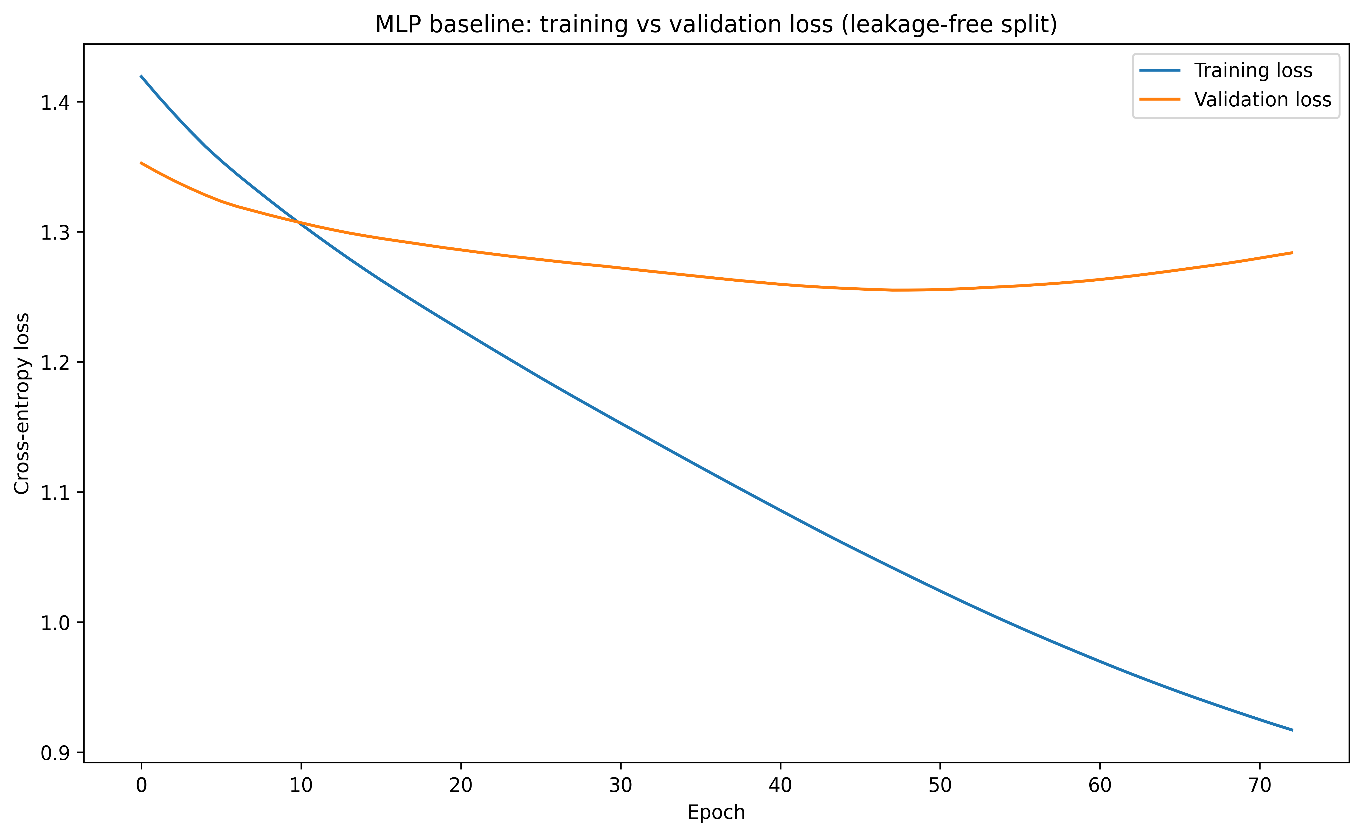


Supplementary Figure S2. Training loss and validation loss


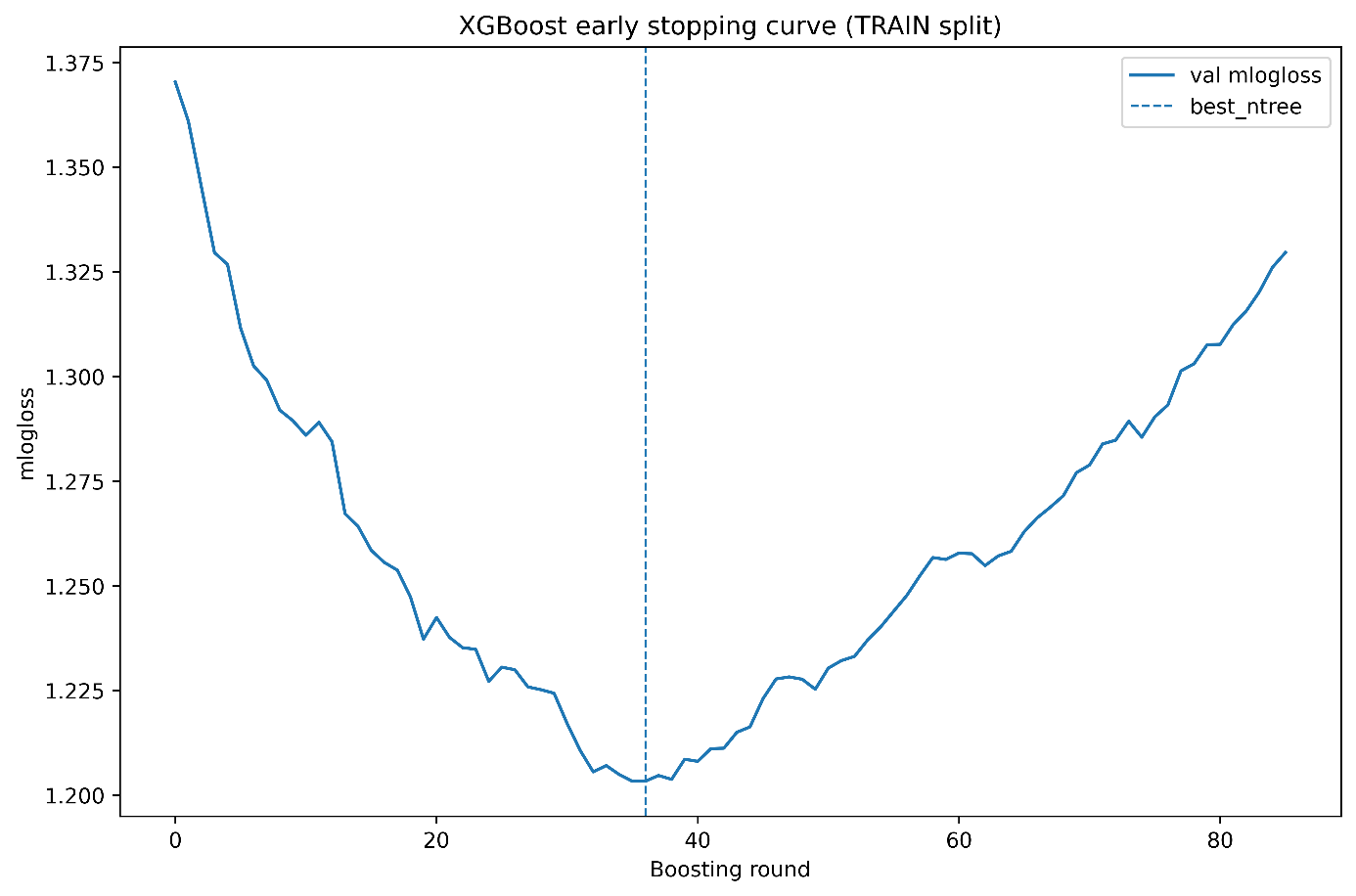


Supplementary Figure S3. XGBoost early-stopping curve on the training split used to select the optimal number of trees under the leakage-free pipeline.

Validation mlogloss decreases until the best iteration (vertical dashed line) and increases afterward, indicating overfitting beyond that point; early stopping selects the optimal number of boosting rounds using only training-split validation data.

Supplementary Table S2. Hyperparameter tuning and early-stopping summary for the tree-based models

| **Model** | **RandomForest** | **GradientBoosting (sklearn)** | **XGBoost (native train)** |
| --- | --- | --- | --- |
| **Early stopping** | N/A | internal (used n_estimators_=106) | native xgb.train (mean_ntree~66, final_best_ntree=36) |
| **Selected hyperparameters** | n_estimators=500; max_depth=10; min_samples_split=5; min_samples_leaf=2 | n_estimators=800; max_depth=3; learning_rate=0.03; subsample=1.0 | max_depth=5; eta=0.05; subsample=0.8; colsample_bytree=0.8; min_child_weight=1.0; lambda=1.0; alpha=0.0; eval_metric=mlogloss; tree_method=hist |
| **CV score (macro recall)** | 0.508 | 0.464 | 0.512 |
| **Accuracy** | 0.486 | 0.514 | 0.459 |
| **Macro Precision** | 0.362 | 0.417 | 0.325 |
| **Macro Recall** | 0.391 | 0.419 | 0.373 |
| **Macro F1** | 0.365 | 0.403 | 0.341 |
| **Weighted Precision** | 0.42 | 0.471 | 0.384 |
| **Weighted Recall** | 0.486 | 0.514 | 0.459 |
| **Weighted F1** | 0.438 | 0.472 | 0.411 |
| **Macro AUC (OvR)** | 0.688 | 0.731 | 0.709 |
| **Recall Class 2** | 0 | 0 | 0 |
| **Recall Class 3** | 0.786 | 0.786 | 0.714 |
